# Real-to-Sim Calibration and Cross-Domain Trajectory Validation of a Low-Cost Multi-Sensor UGV Digital Twin

**DOI:** 10.3390/s26185729

**Published:** 2026-09-09

**Authors:** Carlos Villagomez Alfaro, Zandra Betzabe Rivera Chavez, Marco Claudio De Simone, Domenico Guida

**Affiliations:** 1 MEID4, University Spin-Off and Research Institution for AI-Based Innovation, Via Rosa Jemma 2, 84091 Battipaglia, Italy; villagomez@meid4.it; 2 Department of Industrial Engineering, University of Salerno, Via Giovanni Paolo II, 84084 Fisciano, Italy; mdesimone@unisa.it (M.C.D.S.); guida@unisa.it (D.G.)

**Keywords:** Real-to-Sim calibration, cross-domain trajectory deviation, multi-sensor fusion, EKF, SLAM, digital twin, NVIDIA Isaac Sim, ROS 2, mobile robot navigation

## Abstract

Bridging the real-to-sim gap in low-cost autonomous mobile robotics requires careful cross-domain alignment of kinematic geometry, actuator behavior, and sensor characteristics. This paper presents a systematic Real-to-Sim parameter calibration and multi-stage experimental validation framework for a low-cost differential-drive unmanned ground vehicle (Jackson UGV) operating within NVIDIA Isaac Sim. The calibration process distinguishes initial product/design references, directly measured physical geometry, empirically adjusted ROS 2 runtime parameters, and simulation-specific PhysX parameters. By tuning virtual wheel geometry, inertial sensor profiles, and PhysX joint-drive damping, the proposed framework enables controlled comparison between physical execution and digital-twin behavior. Benchmark evaluations across three experimental stages—square waypoint-tracking trajectories, continuous figure-eight maneuvers, and dynamic obstacle avoidance in a mapped maze course—quantify rotational repeatability, temporal alignment, estimator consistency, and cross-domain trajectory deviation. The square and figure-eight trials reveal a proprioceptive “estimator optimism gap” in which onboard EKF estimates remain internally repeatable while underestimating terminal displacement relative to external floor measurements or simulator-provided reference poses. In Stage 3, 2D LiDAR-based localization and Nav2/DWB local planning reduce dependence on purely proprioceptive dead reckoning, achieving 100% goal completion without observed collision events across both physical and virtual deployments. The results support the calibrated digital twin as a controlled simulation baseline for studying cross-domain navigation behavior and for future sim-to-real evaluation of autonomous mobile robot navigation algorithms.

## 1. Introduction

Physical robotic prototypes often feature prohibitive procurement costs, while affordable hardware platforms typically suffer from systematic sensor latencies, non-linear wheel slippage, and restricted edge-computing budgets, as highlighted by recent industrial benchmarks on embedded autonomy [1,2,3]. Similar affordability and deployment constraints have also been reported in low-cost UGV sensing and agricultural robotics applications, including obstacle-avoidance platforms and modular autonomous vehicles for sustainable viticulture in resource-constrained environments [4,5].

To mitigate physical hardware degradation and accelerate software validation protocols, contemporary robotics frameworks rely extensively on simulation-based testing environments and digital-twin workflows for autonomous mobile robots [6,7,8]. Yet, transferring navigational models from a virtual agent to a physical chassis triggers a foundational scientific challenge known as the “Sim-to-Real gap,” conceptualized by Tobin et al. [9] and expanded in mobile-robot navigation and reinforcement-learning studies [10,11]. Traditional open-source robotics simulators frequently exhibit limitations when modeling real-world dynamic complexities, such as high-frequency surface friction variances, structural sensor measurement drift, and middleware transmission delays [12,13]. Consequently, modern autonomous frameworks require a systematically calibrated digital twin (DT) taxonomy based on the core bidirectional architectures formalized by Grieves and Vickers [14], extended through digital-twin modeling methodologies [15,16], and adapted to robotic and physical-AI paradigms [17,18]. An operational DT framework must systematically synchronize physical hardware state vectors with a high-fidelity virtual engine capable of repeatable physics-based simulation and realistic sensor data synthesis [19], establishing an online verification testbed where simulation and reality complement one another [20].

A common evaluation vulnerability in cross-domain robotics studies involves treating simultaneous localization and mapping (SLAM) pose estimations on a physical platform as absolute ground-truth trajectories. In the absence of high-precision exteroceptive tracking systems, such as overhead motion-capture cameras, directly equating virtual simulator ground-truth coordinates with physical SLAM outputs introduces methodological errors. To address this rigor requirement, cross-domain trajectory evaluations must be structurally formulated to quantify relative cross-domain trajectory deviations rather than unverified absolute spatial errors, preserving scientific modesty and statistical clarity during validation.

This paper addresses these foundational challenges by introducing a systematic calibration and validation methodology deployed on the Jackson UGV platform—a differential-drive mobile robot integrating an NVIDIA Jetson Orin Nano edge processor and an ESP32-S3 microcontroller running over a Robot Operating System 2 (ROS 2 Humble) middleware infrastructure. Rather than focusing on promotional platform showcases, this work investigates the isolation and quantification of low-level mechanical, actuator, and sensory variables to ensure trajectory consistency under classical Navigation2 (Nav2) path-tracking control stacks. The corresponding virtual replica is synthesized within NVIDIA Omniverse Isaac Sim, exploiting the hardware-accelerated PhysX SDK and OmniGraph pipelines to inject empirically derived Gaussian noise parameters directly into the synthetic sensor streams, thereby reducing selected components of the structural reality gap through Real-to-Sim calibration and controlled cross-domain validation protocols [13,21,22].

Several recent studies have addressed complementary aspects of mobile-robot simulation, digital-twin validation, and Sim-to-Real transfer. Rivera et al. [19] investigated physics-based UGV modeling in Gazebo/ROS, while Daza et al. [13] quantified the reality gap between simulated and real vehicle trajectory tracking. More recent studies have exploited NVIDIA Isaac Sim for digital-twin synchronization [6], simulation-based controller optimization [8], and reinforcement-learning-based Sim-to-Real navigation [11]. Bergs et al. [23] further proposed a systematic physical–digital comparison framework for mobile-robot navigation using repeated real-world and Isaac Sim experiments. Table 1 summarizes the methodological differences between these representative studies and the present work, with particular emphasis on physical parameter identification, simulator-specific calibration, repeated cross-domain validation, and sensitivity/generalization analysis.

As shown in Table 1, the novelty of the present work does not arise from digital-twin construction or physical–virtual navigation comparison alone, since these elements have been addressed individually in prior studies. The methodological distinction lies in their combined treatment within a low-cost multi-sensor UGV framework. Specifically, the proposed workflow separates directly measured physical geometry and runtime odometry calibration from simulator-specific effective parameters; evaluates estimator behavior against independent physical endpoint measurements and simulator-provided ground truth across three complementary experimental stages; and subsequently quantifies how wheel-geometry, PhysX, obstacle-layout, surface, and LiDAR perturbations propagate to trajectory deviation, obstacle clearance, navigation time, and task completion. This combination provides a parameter-level Real-to-Sim validation framework rather than solely a robot-modeling, controller-optimization, policy-transfer, or navigation-prediction study.

The primary contributions of this research are formulated through four modest but clear validation vectors:

1. A reproducible, sensor-based ROS 2 and NVIDIA Isaac Sim calibration workflow customized for a low-cost, multi-sensor UGV framework.
2. An empirical Real-to-Sim system identification methodology that isolates effective wheel radius, effective track width, serial communication transport delay, and sensor noise characteristics.
3. A cross-domain validation protocol evaluating tracking consistency through systematic square, figure-8, and obstacle-course trajectories.
4. A quantitative performance evaluation based on path deviation, localized heading drift, occupancy grid map structural similarity indices (SSIM), system latency, and execution repeatability across multiple trial distributions.

Because the contribution of this study is a Real-to-Sim calibration and validation framework rather than a new path-planning or control algorithm, a direct numerical ranking against MPPI-, reinforcement-learning-, or other planner-specific methods would not provide an equivalent comparison. Such results depend strongly on robot geometry, sensing, map structure, velocity limits, controller tuning, and evaluation protocol. Accordingly, Table 1 compares the proposed framework with representative state-of-the-art approaches at the methodological level. Within the experimental study itself, the navigation controller is deliberately held fixed through ROS 2 Nav2/DWB so that the measured differences can be attributed to physical–virtual calibration, state estimation, and controlled parameter or environmental perturbations rather than to changes in the planning algorithm.

The remainder of this manuscript is organized as follows: Section 2 details the Materials and Methods, encompassing the dual-tier physical hardware architecture, digital twin URDF structure, and multi-rate ROS 2 middleware topology. Section 2.4 establishes the mathematical formulations for the system identification and parameter calibration protocols. Section 3 presents the empirical multi-trajectory experimental results and statistical distributions. Section 4 analyzes the cross-domain deviations, control latencies, and localized deadbands, while Section 5 summarizes the findings and outlines directions for future learning-based development.

## 2. Materials and Methods

### 2.1. Physical Platform and Real-to-Sim Data Logging Architecture

The physical foundation of the experimental architecture relies on an affordable, custom-engineered unmanned ground vehicle (UGV), designated as the Jackson UGV. Within the broader research ecosystem, the mobile chassis itself is designated as Jackson—governing core differential-drive locomotion, spatial sensing, and navigation telemetry—while Lucas represents the auxiliary audio-perception and voice interface layer for interactive edge AI. Because audio perception is decoupled from motion control, all kinematic calibration, multi-sensor fusion, and cross-domain trajectory validation experiments reported in this work are performed exclusively on the locomotion and sensing hardware of the Jackson UGV.

To support repeatable real-time execution and edge-computing efficiency, the hardware architecture implements an asynchronous dual-tier topology, separating low-level embedded kinematic processing from high-level ROS 2 middleware routing, state estimation, navigation, and sensor telemetry aggregation. The ESP32-S3 N16R8 Gold Edition (Lonely Binary, Sydney, Australia) operates as an embedded sensor-acquisition and communication unit. Its firmware acquires quadrature-encoder counts and MPU-6050 inertial measurements from a SunFounder MPU-6050 sensor module (SunFounder, Shenzhen, China), packages the data, and transmits them to the Jetson computer for wheel-odometry computation, state estimation, navigation, and motor control. The physical chassis uses a non-holonomic differential-drive configuration powered by two DC 6V gear motors operating at a nominal 100RPM with 66mm diameter rubber wheels. Motor commands generated by the Jetson are sent through an I^2^C interface to a generic PCA9685 PWM module based on the PCA9685 controller IC (NXP Semiconductors, Eindhoven, The Netherlands), which provides the PWM signals for the motor-control stage, followed by a generic TB6612FNG dual H-bridge motor-driver module based on the TB6612FNG IC (Toshiba, Kawasaki, Japan).

High-level processing, spatial perception, state estimation, and real-time data logging are offloaded to an NVIDIA Jetson Orin Nano Super Developer Kit with an 8GB Jetson Orin Nano module (NVIDIA Corporation, Santa Clara, CA, USA). The Jetson runs Ubuntu 22.04 LTS and ROS 2 Humble over a Fast DDS communications backbone. Encoder and IMU data acquired by the ESP32-S3 are transmitted to the Jetson Orin Nano through a dedicated high-speed serial link using a checksum-validated byte-packet structure to preserve frame integrity. Motor commands follow a separate actuation path from the Jetson through the I^2^C-connected PCA9685 module to the TB6612FNG motor driver.

A critical engineering feature for Real-to-Sim logging is the isolated dual-tier power distribution framework. High-frequency actuator switching and DC motor transients can introduce electromagnetic interference (EMI), voltage drops, and micro-reset events that degrade sensor reliability. To mitigate these effects, the power architecture separates the computational rail from the actuation rail. The primary computing infrastructure is powered by a 145W UGREEN PB205 25,000mAh USB-C Power Delivery (PD) power bank (UGREEN, Shenzhen, China), while the motor drivers and DC actuators are powered by an independent rail comprising five 1.2V NiMH AA rechargeable batteries. This electrical isolation helps preserve clean telemetry streams for ROS 2 bag recording.

The physical sensor suite mounted on the Jackson UGV provides continuous spatial and inertial awareness. Planar environment mapping and obstacle detection are performed using a 2D $360^{\circ }$ RPLIDAR A1M8 sensor (Shanghai Slamtec Co., Ltd., Shanghai, China) with a nominal scanning radius of 12m, connected to the Jetson edge computer through USB. Inertial state vectors, including 3-axis linear accelerations and angular velocities, are acquired by an MPU-6050 (GY-521) 6-DOF inertial measurement unit (SunFounder, Shenzhen, China) communicating with the ESP32-S3 over a local hardware I^2^C bus at 400kHz. Time-of-flight (ToF) range sensors are included in the physical hardware layout as auxiliary proximity sensors; however, the validation experiments reported in this work rely on wheel encoders, the MPU-6050 IMU, and the RPLIDAR A1M8. ToF measurements are not used in the quantitative trajectory or navigation metrics.

Visual perception is available through a dual-camera setup consisting of a Yahboom 8MP IMX219 CSI camera (Yahboom, Shenzhen, China) fitted with a $160^{\circ }$ ultra-wide-angle lens and a Teros TE-9072 2K USB webcam (TEROS, Shenzhen, China). These cameras are included as optional perception hardware for future vision-based navigation extensions, but they are not used in the quantitative trajectory-validation metrics reported in this study.

**Figure 1:**
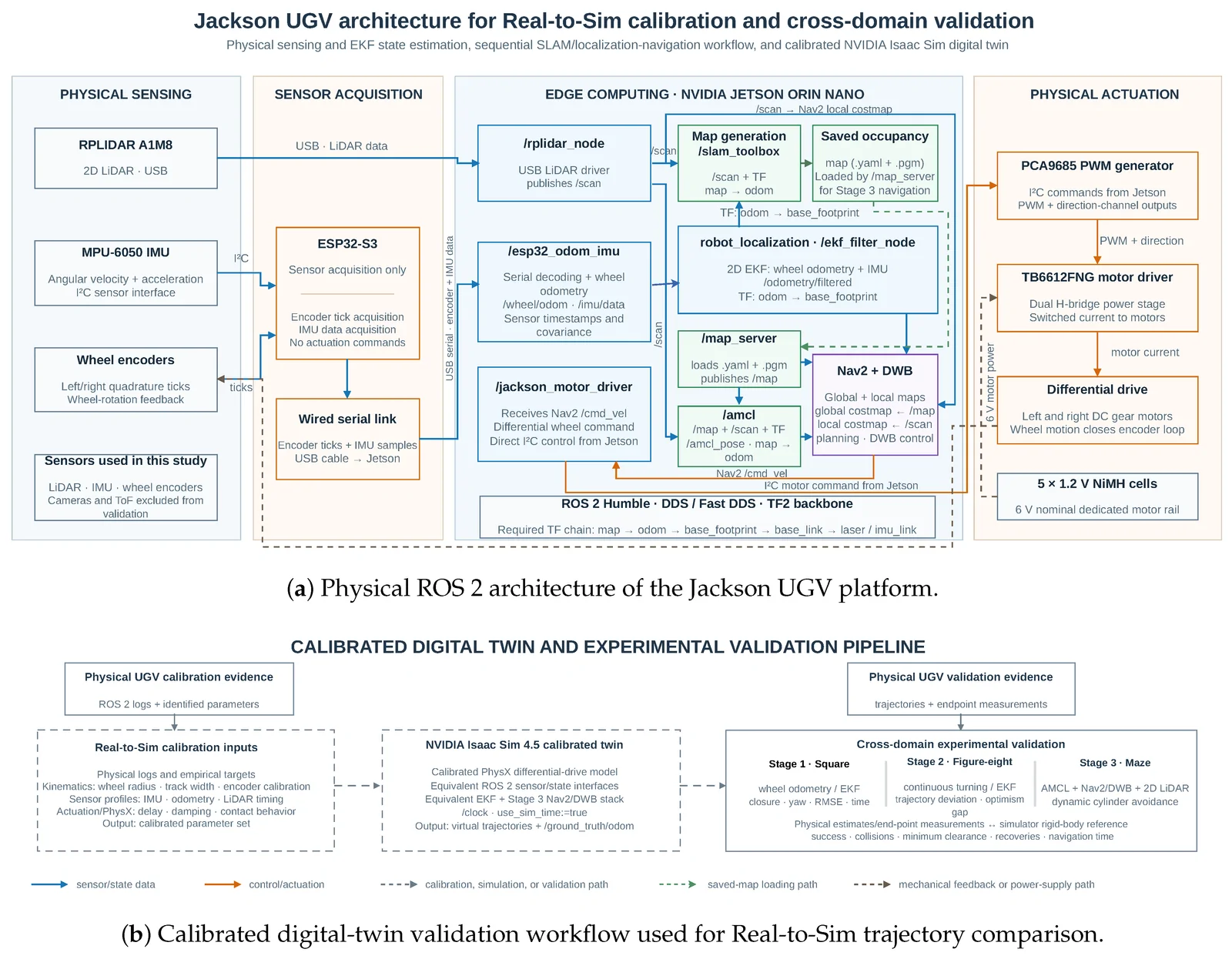
Jackson UGV Real-to-Sim architecture. (**a**) Physical ROS 2 sensing, state-estimation, navigation, and actuation architecture. (**b**) Calibrated Isaac Sim digital-twin workflow for Real-to-Sim parameter mapping and cross-domain validation.

As summarized in Figure 1a, the physical platform separates sensor acquisition, embedded actuation, ROS 2 state estimation, and Nav2 navigation into modular layers. Raw encoder ticks, IMU measurements, LiDAR scans, transform frames, odometry estimates, and command topics are recorded into ROS 2 bag files for subsequent calibration and validation.

To map the real physical parameters into the virtual replica, a continuous Real-to-Sim data logging pipeline is executed natively within ROS 2. The recorded telemetry includes raw encoder ticks, IMU orientation and angular-velocity data, LiDAR scan arrays, odometry estimates, TF transformations, and velocity-command topics. High-bandwidth sensor streams such as /scan and /imu/data use low-latency QoS settings, while control and state-estimation topics such as /cmd_vel, /wheel/odom, and /odometry/filtered use reliable delivery with bounded history depth, as detailed in Appendix C. This logging structure provides a repeatable baseline dataset for physical parameter identification and virtual asset alignment.

Figure 1b illustrates how the physical ROS 2 logs support calibration of the Isaac Sim digital twin and subsequent cross-domain validation. The simulator provides reference pose information through /ground_truth/odom, while the physical platform relies on onboard state-estimation sources such as /odometry/filtered and AMCL pose estimates. Consequently, the validation framework reports cross-domain trajectory deviations rather than treating physical SLAM or AMCL outputs as absolute ground truth.

**Figure 2:**
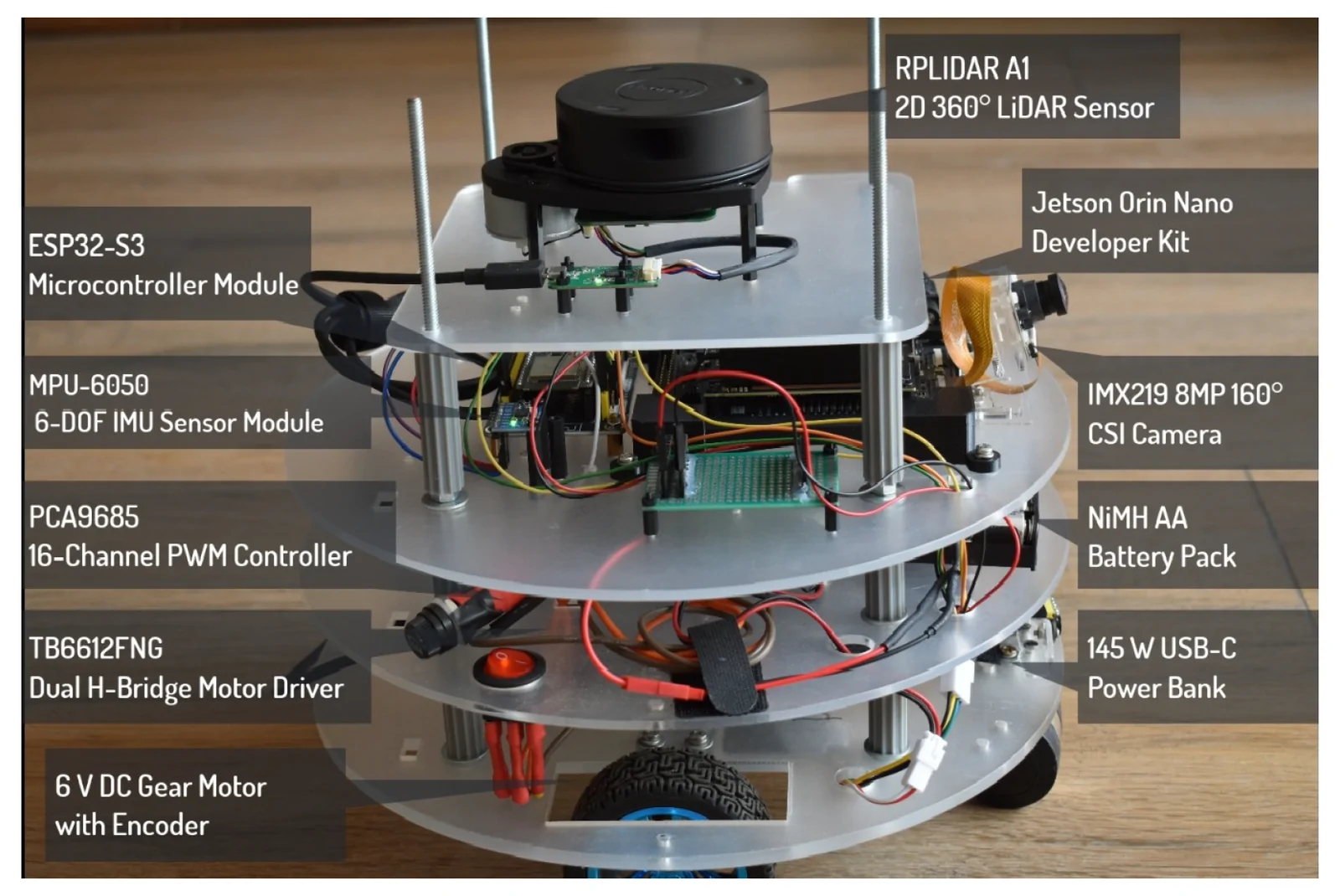
Physical implementation of the Jackson UGV chassis showing the spatial arrangement of the RPLIDAR A1M8, edge computing components, and isolated battery assemblies.

The physical implementation of the Jackson UGV and the spatial arrangement of its main hardware components are shown in Figure 2.

### 2.2. Digital Twin Construction and Sensor Synthesis

**Figure 3:**
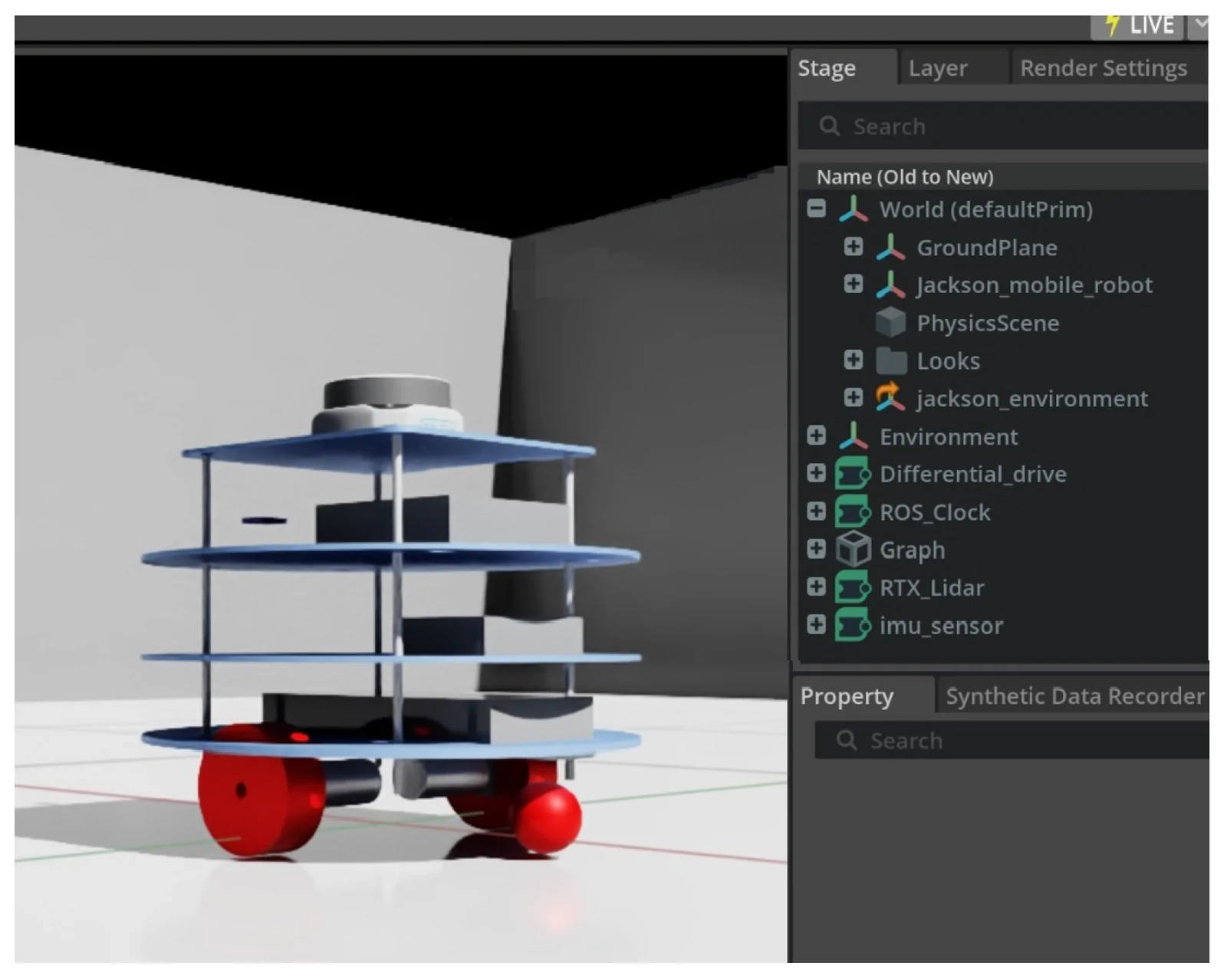
Digital twin implementation of the Jackson UGV within NVIDIA Isaac Sim. The multi-panel view establishes the architectural mapping defined in Section 2.2, showcasing the physical multi-tier chassis layout (left) alongside the explicit PhysX environment hierarchy, hardware-accelerated RTX_Lidar array, and stochastic imu_sensor nodes (right).

The cross-domain replication framework requires a precise mapping of mechanical properties, kinematic chains, and sensor configurations. To achieve this, a high-fidelity digital twin of the Jackson UGV was constructed within the NVIDIA Omniverse Isaac Sim environment. As illustrated in Figure 3, the simulation workspace explicitly couples the visual and collision geometries with the underlying physics layer, initializing the programmatic foundation for the ray-traced perception and rigid-body dynamics detailed below.

#### 2.2.1. Kinematic Modeling and URDF Structure

To evaluate cross-domain state consistency between the virtual and physical domains, the digital twin of the UGV platform is initialized through a geometric and kinematic representation in a Unified Robot Description Format (URDF) model. The multi-tier chassis, dual-drive wheels, caster support, and sensor mounts are represented as a highly detailed rigid-body tree structure anchored to a zero-elevation ground projection designated as base_footprint, which maintains a rigid spatial transform to the primary mechanical coordinate origin, base_link.

The kinematic transformation maps joint-space wheel angular velocities to the task-space navigation frame. Assuming planar motion under non-holonomic constraints, the ideal kinematics of the robot base frame (base_link) relative to the odometric frame (odom) are modeled as:

(1) $$\left[\begin{array}{c} \dot{x}\\ \dot{y}\\ \dot{\theta } \end{array}\right]=\left[\begin{array}{cc} \cos\theta & 0\\ \sin\theta & 0\\ 0 & 1 \end{array}\right]\left[\begin{array}{c} v\\ \omega \end{array}\right]$$

where *x*, *y*, and θ denote the planar coordinates and heading angle, while *v* and ω represent the linear and angular velocities. Under nominal conditions, these task-space velocities are related to the left ($\omega_{L}$) and right ($\omega_{R}$) wheel angular velocities by:

(2) $$v=\frac{r_{0}}{2}\left(\omega_{R}+\omega_{L}\right)$$

(3) $$\omega =\frac{r_{0}}{B_{0}}\left(\omega_{R}-\omega_{L}\right)$$

where $r_{0}=0.033\;m$ is the directly measured physical wheel radius, obtained from the approximately 66mm outer diameter of the installed wheels, and $B_{0}=0.198\;m$ is the documented physical wheel-separation baseline. An earlier wheel-radius value of 0.034m taken from the product specification was superseded by the direct physical measurement. These values define the physical geometric reference, while the encoder-to-distance conversion and the simulator-specific effective parameters are calibrated separately in Section 2.4. Physical component distribution is organized into a four-level vertical hierarchy (Actuation, Power, Compute, and Perception decks). Mass distributions and principal inertia tensors ($I_{xx},I_{yy},I_{zz}$) were extracted directly from structural CAD assemblies. Complete deck descriptions, frame transformation chains (tf2), and cross-domain frame naming mappings (lidar_link vs. laser) are provided in Appendix B.

#### 2.2.2. Rigid-Body Dynamics and Physics Properties

Deterministic dynamic simulation within the NVIDIA Omniverse Isaac Sim environment relies on the precise propagation of empirical inertial, material, and surface contact properties into the integrated PhysX SDK engine [12]. Rather than relying on idealized primitives, mass distributions, localized centers of mass (CoM), and principal inertia tensors were explicitly derived from the structural CAD assemblies based on component material densities (e.g., PMMA acrylic tiers, copper motor windings, and lithium-ion/NiMH battery chemical cores). For each discrete rigid link *i* within the UGV tree structure, the diagonalized rotational inertia tensor $I_{i}$ relative to its localized CoM coordinate frame is formulated as

(4) $$I_{i}=\left[\begin{array}{ccc} I_{xx} & 0 & 0\\ 0 & I_{yy} & 0\\ 0 & 0 & I_{zz} \end{array}\right]$$

The spatial aggregation of these tensors establishes a representative global center of mass, preventing the emergence of artificial stability anomalies during aggressive angular acceleration profiles or sudden command transitions.

To accurately emulate the non-holonomic velocity bounds of the differential-drive architecture, the physical interface between the molded rubber wheels and the driving substrate is governed by an anisotropic Coulomb friction model implemented within the PhysX solver [13]. This configuration isolates the friction characteristics into orthogonal components along the longitudinal (tangential travel) and lateral (lateral slip) slip axes. The boundary limits of the friction envelope are tightly bounded by independent static ($\mu_{s}$) and dynamic ($\mu_{d}$) friction coefficients:

(5) $$f_{\mathrm{friction}}\leq \left\{\begin{array}{cc} \mu_{s}f_{\mathrm{normal}} & \mathrm{under}\;\mathrm{stick}\;\mathrm{conditions}\\ \mu_{d}f_{\mathrm{normal}} & \mathrm{under}\;\mathrm{slip}\;\mathrm{conditions} \end{array}\right.$$

The friction parameters were treated as part of the simulation-specific PhysX configuration and evaluated indirectly through the trajectory-level validation metrics reported in Section 3.

#### 2.2.3. Actuator Dynamics and PhysX Joint Drive Configuration

The physical command pipeline operates at a 20Hz control frequency, with a command timeout of 0.60s in the documented hardware configuration. In the present work, the physical actuator chain is not modeled through an independently identified electromechanical transfer function. Instead, the digital twin uses actuator-alignment parameters to stabilize the response of the virtual wheel joints under repeated velocity commands and to reproduce the observed navigation behavior at the trajectory level.

In the general actuator-dynamics configuration documented for jackson_robot_july.usd, the PhysX joint-drive parameters were specified with zero position stiffness (K=0.0N·m/rad) and a damping coefficient of D=3000.0N·m·s/rad. The zero-stiffness setting avoids artificial spring-like restoring torques during velocity-controlled wheel motion, while the damping value acts as a numerical velocity-tracking regularization factor inside the PhysX solver integrated in NVIDIA Isaac Sim 4.5.0 and reduces high-frequency velocity chatter during continuous command updates.

For the Stage 3 closed-loop sensitivity campaign, the active PhysX angular-drive damping was independently verified at $D_{0}=10,000$, with the perturbed conditions D=9000 and D=11,000. Therefore, D=3000 should not be interpreted as the Stage 3 operating-point value; it refers to the general actuator-dynamics configuration, whereas $D_{0}=10,000$ defines the active Stage 3 baseline used for the local sensitivity analysis. The Stage 3 damping perturbations modified the effective joint-drive parameter only, and the wheel collision meshes were not geometrically resized.

The physical control loop also includes practical command-shaping elements, including PWM deadband compensation and acceleration limiting. These mechanisms affect the measured physical response, but they are treated here as part of the documented physical runtime behavior rather than as parameters of a fully identified motor model. Consequently, actuator alignment in this study is validated indirectly through trajectory-level agreement across the square, figure-eight, and dynamic-obstacle experiments, while detailed motor step-response identification is left for future work.

#### 2.2.4. RTX Ray Tracing and PhysX Sensor Emulation

To evaluate local navigation stacks under matching sensory conditions, exteroceptive and proprioceptive sensors are emulated using hardware-accelerated RTX Ray Tracing and PhysX state extraction pipelines [21].

##### LiDAR Sensor Emulation

The onboard SLAMTEC RPLIDAR A1M8 (SLAMTEC Co., Ltd., Shanghai, China) planar scanner is replicated using the simulator’s hardware-accelerated RTX-based Lidar utility. Rather than relying on generic manufacturer bounds, the virtual sensor is configured using direct measurements captured from the physical ROS 2 /scan topic, which reveals a true scan period of 0.13465s (an operational frequency of 7.43Hz) and an angular increment of 0.005807rad (0.3327°). This layout yields approximately 1080 samples per full rotation over a 0.15m to 12.0m operational range. The resulting synthetic range vector $d_{L}$ is published directly to the middleware layer to match real-world data structures:

(6) $$d_{L}={\left[d_{1},d_{2},\ldots ,d_{N}\right]}^{T}$$

To approximate physical ranging uncertainties during initial indoor mapping simulations, the range values are injected with zero-mean Gaussian white noise assuming a fixed standard deviation of $\sigma_{\mathrm{LiDAR}}=0.010\;m$. While the hardware retains its 12.0m maximum limit, both the physical and virtual SLAM pipelines enforce a software processing constraint capped at 8.0m for algorithmic evaluation.

##### Inertial Measurement Unit (IMU) Emulation

Sensor feedback for the MPU-6050 6-DOF IMU is synthesized by extracting the linear accelerations and angular velocities of the designated imu_link within the PhysX solver. To model physical sensor uncertainty and bias variations, the raw simulated outputs are modified by incorporating configurable noise and drift terms [22]:

(7) $$a_{\mathrm{sim}}=a_{\mathrm{true}}+b_{a}+\eta_{a}$$

(8) $$\omega_{\mathrm{sim}}=\omega_{\mathrm{true}}+b_{\omega }+\eta_{\omega }$$

where $b_{a}$ and $b_{\omega }$ represent localized sensor bias offsets, and $\eta_{a}$, $\eta_{\omega }$ represent zero-mean Gaussian white noise components. Initial noise-density boundaries are derived from the manufacturer’s datasheet specifications ($0.005{}^{\circ}/s/\sqrt{\mathrm{Hz}}$ for the gyroscope and $400\;\mu g/\sqrt{\mathrm{Hz}}$ for the accelerometer). At the hardware’s configured 21Hz digital low-pass filter (DLPF) bandwidth, these profiles yield an initial standard deviation of $3.999\times 10^{-4}\;\mathrm{rad}/s$ for angular velocity and $1.7976\times 10^{-2}\;{m/s}^{2}$ for linear acceleration to initialize the measurement noise matrix.

The synthesized sensor outputs are handled by high-frequency OmniGraph pipelines that bridge the internal simulator state with native ROS 2 nodes, publishing standardized topics (/scan, /imu, and /odom) using Fast DDS middleware. This structural mapping ensures that the path planning and localization stacks process identical message data types during both virtual evaluation and physical deployment.

### 2.3. Multi-Sensor Data Processing and ROS 2 Middleware Topology

#### 2.3.1. Asynchronous Multi-Rate Sensor Ingestion and Fusion

To manage state estimation within the onboard processing constraints of the edge-computing hardware, the UGV architecture uses an asynchronous, multi-rate sensor-ingestion pipeline. Proprioceptive and exteroceptive data streams are sampled at distinct operational frequencies according to their hardware interfaces and noise characteristics:

- MPU-6050 IMU: Streamed at a 20Hz publication rate with an internal 21Hz digital low-pass filter configuration.
- Quadrature encoders: Sampled at a 20Hz discrete update rate, matching the processing interval Δt=0.05s.
- RPLIDAR A1M8: Ingested at a measured 7.43Hz sweep frequency determined by the hardware rotational period.

Dead-reckoning quantities are calculated from the raw quadrature-encoder transitions. Let $N_{L}$ and $N_{R}$ denote the accumulated pulses during the discrete sampling interval Δt=0.05s. The corresponding left- and right-wheel displacements, $\Delta s_{L}$ and $\Delta s_{R}$, are

(9) $$\Delta s_{L}=2\pi r_{\mathrm{run}}\frac{N_{L}}{C_{e,\mathrm{run}}},\;\Delta s_{R}=2\pi r_{\mathrm{run}}\frac{N_{R}}{C_{e,\mathrm{run}}},$$

where $r_{\mathrm{run}}=0.033\;m$ is the documented runtime wheel-radius parameter and $C_{e,\mathrm{run}}=621\;\mathrm{TPR}$ is the active calibrated encoder-resolution parameter used by the physical wheel-odometry driver.

State estimation was implemented with the ROS 2 Humble robot_localization package (version 3.5.4). The EKF ran at 30Hz in planar mode, with a sensor timeout of 0.20s and odom as its world frame. It published the transform from odom to base_footprint, together with the filtered state on /odometry/filtered.

The filter uses the standard 15-component state representation of robot_localization. This representation comprises three-dimensional position (x,y,z), orientation (ϕ,θ,ψ), linear velocity $\left(v_{x},v_{y},v_{z}\right)$, angular velocity $\left(\omega_{x},\omega_{y},\omega_{z}\right)$, and linear acceleration $\left(a_{x},a_{y},a_{z}\right)$. Under the planar configuration, the non-planar components are constrained by two_d_mode.

Only the variables enabled in the input configurations were fused during the experiments. Encoder-derived /wheel/odom contributed the forward velocity $v_{x}$ and the non-holonomic lateral-velocity constraint $v_{y}=0$, whereas /imu/data contributed only the yaw rate $\omega_{z}$. IMU orientation and linear acceleration were not fused.

The effective measurement variances were $R_{v_{x}}=0.01\;{\left(m/s\right)}^{2}$, $R_{v_{y}}=0.001\;{\left(m/s\right)}^{2}$, and $R_{\omega_{z}}=0.0004\;{\left(\mathrm{rad}/s\right)}^{2}$. The principal EKF settings used in the physical experiments are summarized in Table 2.

RPLIDAR measurements were used by SLAM during mapping and by AMCL during localization; they were not fused directly by the EKF. This separation preserves the distinction between proprioceptive state estimation and exteroceptive localization within the ROS 2 navigation architecture.

#### 2.3.2. Coordinate Frame Topology (tf2) and Cross-Domain Discrepancies

Spatial transformations across the virtual and physical domains are managed using the ROS 2 tf2 library. The structural transformation chain follows the kinematic hierarchy:

(10) $$\begin{array}{cc} \mathrm{map}⟶\mathrm{odom} & ⟶\mathrm{base}\_\mathrm{footprint}⟶\mathrm{base}\_\mathrm{link}\\ \; & ⟶\{\mathrm{laser},\mathrm{imu}\_\mathrm{link},\mathrm{left}\_\mathrm{wheel}\_\mathrm{link},\mathrm{right}\_\mathrm{wheel}\_\mathrm{link}\} \end{array}$$

**Figure 4:**
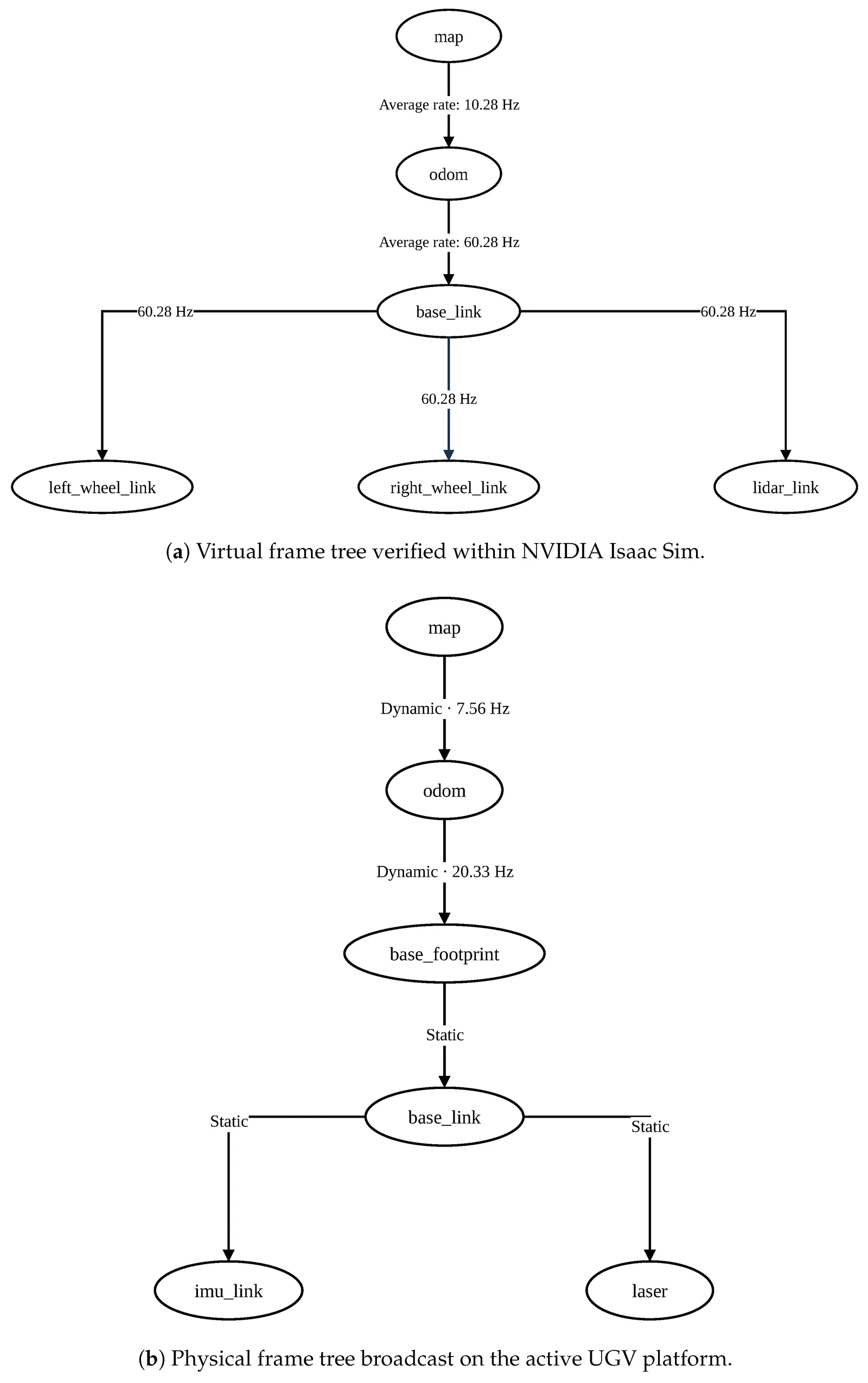
Comparative analysis of the ROS 2 tf2 frame topology. The virtual frame tree (**a**) represents the fully synchronized model inside the simulation, while the physical platform tree (**b**) illustrates the active transformations broadcast during simultaneous localization and mapping (SLAM) execution.

Cross-domain coordinate tree verification is executed to log topological differences between the environments. Figure 4 displays the comparative vertical stack generated by the view_frames utility.

An evaluation of the virtual and physical topologies highlights specific structural variations that affect tracking analysis:

- Sensor Frame Naming: The digital twin (Figure 4a) labels the exteroceptive sensor link as lidar_link, whereas the physical implementation (Figure 4b) registers the frame identifier as laser. This naming offset is corrected by applying a topic remapping layer within the physical initialization launch files.
- Kinematic Projection Reference (base_footprint): The physical architecture explicitly includes a base_footprint link projected onto the ground plane directly below the center of mass, isolating planar translations from high-frequency vertical vibration components. In the simulation, the simplified URDF tree routes transformations from odom directly to base_link to save physics solver resources.
- Wheel Transform Methods: The simulated joint frames (left_wheel_link, right_wheel_link) are updated as active transform branches at 60Hz to track contact mechanics. On the physical UGV, wheel rotations are parsed through discrete /joint_states messages to lower microcontroller communication overhead.

As shown in Figure 4b, the physical SLAM node broadcasts the transform from map to odom at 10Hz, while the EKF localization loop maintains the odom to base_footprint relation at 30Hz. Because external motion-capture tracking was not available, physical poses are treated as stage-dependent reference estimates rather than absolute ground truth: EKF-filtered odometry is used for low-level trajectory trials, and AMCL/SLAM map-frame estimates are used for navigation trials. Discrepancies between virtual and real-world paths are therefore reported as cross-domain trajectory deviations rather than absolute physical tracking errors.

#### 2.3.3. ROS 2 Middleware and Quality of Service (QoS) Optimization

Communication across the onboard edge hardware and external logging workstations is managed via eProsima Fast DDS over ROS 2 Humble. Quality of service (QoS) profiles are strategically configured based on data urgency and bandwidth constraints. High-frequency sensor streams (/scan at 7.43Hz and /imu at 20Hz) enforce a Best Effort reliability policy to eliminate queue latency, whereas critical control and state estimation topics (/cmd_vel and /odom) enforce a Reliable policy with bounded history depth. The complete Fast DDS QoS policy matrix across all active ROS 2 topics is detailed in Appendix C.

### 2.4. Real-to-Sim System Identification and Physical Calibration

To reduce the real-to-simulation domain gap, an empirical system-identification protocol was used to characterize the Jackson UGV differential-drive platform and configure the corresponding NVIDIA Isaac Sim digital twin (jackson_robot_july.usd). The calibration process explicitly distinguishes four parameter categories: initial product and encoder references, directly measured physical geometry, empirically adjusted physical runtime parameters, and simulation-specific effective parameters required to reproduce the virtual robot behavior under PhysX contact dynamics.

#### 2.4.1. Kinematic Parameter Identification

Payload loading, tire compliance, caster interaction, encoder scaling, and floor-contact effects can make the effective floor displacement deviate from nominal mechanical geometry. For methodological clarity, the following four parameter categories were considered:

- Initial product and encoder references: An early wheel-radius value of approximately 0.034m was taken from the seller/product specification during the initial platform configuration. This value was not obtained from the experimental calibration procedure and was subsequently superseded by direct measurement of the installed wheels. The initial encoder-resolution reference was 652TPR.
- Directly measured physical geometry: The installed rubber wheels were measured manually using a measuring tape, yielding an outer diameter of approximately 66mm and therefore a physical wheel radius of $r_{\mathrm{meas}}=0.033\;m$. The documented wheel separation used by the physical platform was $B_{\mathrm{run}}=0.198\;m$.
- Documented physical ROS 2 runtime configuration: The physical wheel-odometry implementation used $r_{\mathrm{run}}=0.033\;m$, $B_{\mathrm{run}}=0.198\;m$, and an active encoder-resolution parameter of 621TPR. The floor-translation experiments were used to refine the encoder-to-distance conversion through the TPR parameter rather than to estimate a different physical wheel radius. The detailed physical measurement and calibration derivations are provided in Appendix A.
- Isaac Sim V16/V17 digital-twin parameters: The virtual values $r_{\mathrm{sim}}=0.031650\;m$ and $B_{\mathrm{sim}}=0.210481\;m$ are simulator-specific effective parameters used to align virtual odometry and EKF behavior with PhysX rigid-body motion. They are not physical measurements of the Jackson UGV.

This distinction prevents conflating early product references, directly measured physical geometry, empirically adjusted runtime odometry parameters, and simulator-specific effective parameters. The physical experiments validate the behavior of the platform and its EKF-based state-estimation pipeline, while the virtual parameters are specific to Isaac Sim and compensate for simulator-dependent contact behavior, joint-state integration, and simulated wheel–ground interaction.

A separate parameter scope applies to the Stage 3 closed-loop navigation campaign. The active Stage 3 differential-controller and joint-state odometry configuration used $r_{0}=0.03300\;m$ and $B_{0}=0.19800\;m$. These values define the operating point perturbed in the local sensitivity analysis of Section 2.5.6 and should not be conflated with the V16/V17 effective kinematic parameters $r_{\mathrm{sim}}$ and $B_{\mathrm{sim}}$. The Stage 3 perturbations modify the effective controller and odometry geometry; the wheel collision meshes themselves were not geometrically resized.

#### 2.4.2. Actuator Dynamics and PhysX Joint Drive Alignment

The physical command pipeline operates at a 20Hz control frequency, with a command timeout of 0.60s in the documented hardware configuration. Rather than claiming a fully identified electromechanical motor transfer function, the simulation model uses actuator-alignment parameters to stabilize the response of the virtual wheel joints under repeated velocity commands.

In jackson_robot_july.usd, the PhysX joint-drive parameters were configured with zero position stiffness (K=0.0N·m/rad) and a damping coefficient of D=3000.0N·m·s/rad. This damping value is interpreted as a numerical velocity-tracking regularization factor inside the PhysX solver. It reduces high-frequency velocity chatter and avoids artificial spring-like restoring forces during continuous wheel-velocity updates. The separate Stage 3 navigation operating point used $D_{0}=10,000$, as documented in Section 2.2.3 and Section 2.5.6.

#### 2.4.3. Proprioceptive Sensor Calibration

The physical platform publishes encoder-derived odometry on /wheel/odom and inertial measurements on /imu/data. These signals are fused by the ROS 2 EKF to produce /odometry/filtered. Static IMU profiling and hardware auditing were used to improve the consistency of yaw-rate integration and gravity alignment across physical and virtual domains.

- Yaw-rate convention: The physical IMU yaw-rate sign was audited to ensure consistency with ROS 2 planar-frame conventions. The objective was to maintain a standard counter-clockwise positive yaw convention across the physical and virtual pipelines.
- Gravity scaling: Static acceleration measurements were compared against standard gravity. A measured static acceleration of approximately $9.299\;{m/s}^{2}$ was scaled by approximately 1.054× to align the physical IMU output with $9.810\;{m/s}^{2}$.
- Virtual yaw scaling: The Isaac Sim V16/V17 configuration used an empirical angular-velocity scaling factor of 0.6953 in the virtual IMU pipeline to improve yaw-rate consistency between joint-state odometry, EKF output, and PhysX ground-truth reference.

#### 2.4.4. Parameter Mapping Matrix

Table 3 summarizes the parameter stratification used throughout the Real-to-Sim calibration workflow. The table distinguishes initial product/design references, documented physical ROS 2 runtime parameters, direct physical measurements and calibration evidence, and simulation-specific effective parameters implemented in the Isaac Sim V16/V17 digital twin.

An early wheel-radius value of approximately 0.034m originated from the seller/product specification and was subsequently superseded by direct measurement of the installed wheels, which yielded a physical wheel radius of $r_{\mathrm{meas}}=0.033\;m$. The physical wheel-odometry runtime configuration retained $r_{\mathrm{run}}=0.033\;m$ and $B_{\mathrm{run}}=0.198\;m$, while the floor-translation calibration was applied through the encoder-resolution parameter, changing the initial reference of 652TPR to the active runtime value of 621TPR. The $360^{\circ }$ rotation consistency check using $r_{\mathrm{meas}}=0.033\;m$ yielded $B_{\mathrm{check}}\approx 0.198\;m$, consistent with the documented physical wheel separation.

The Isaac Sim V16/V17 digital twin uses separate simulator-specific effective parameters, $r_{\mathrm{sim}}=0.031650\;m$ and $B_{\mathrm{sim}}=0.210481\;m$, together with joint-state odometry and virtual IMU scaling. This separation prevents conflating early product references, directly measured physical geometry, runtime odometry calibration, and simulator-specific effective parameters.

### 2.5. Path Tracking Control and Cross-Domain Validation Protocols

To execute a comparative evaluation of the Jackson UGV, the virtual and physical agents are configured with an identical autonomous navigation stack. This section outlines the path planning control parameters, the multi-trajectory experimental profiles, the alignment routines, and the statistical metrics utilized to analyze cross-domain trajectory deviations.

#### 2.5.1. Autonomous Navigation Control Stack

To systematically evaluate platform kinematics, state estimation, and reactive control across increasing operational complexity, the control stack is structured into three experimental regimens:

- Stage 1 (Discrete Square Trajectory): Discrete open-loop and EKF-assisted geometric tracking along a nominal 1.0m×1.0m square profile are governed by a deterministic waypoint controller. This controller enforces explicit velocity deceleration bounds and closed-loop final heading alignment at pivot corners to evaluate localized orientation convergence.
- Stage 2 (Continuous Non-Holonomic Figure-Eight): Curvilinear drift dynamics are evaluated using a continuous velocity-profile generator that commands a constant linear speed (v=0.08m/s) and alternating angular rates (ω=±0.16rad/s) across two nominal R=0.50m loops without intermediate deceleration stops.
- Stage 3 (Closed-Loop Exteroceptive Maze Navigation): Autonomous path planning and dynamic obstacle avoidance are managed via the Robot Operating System 2 (ROS 2) Navigation2 (Nav2) stack. Global paths are generated using the nav2_planner server executing an $A^{*}$ search algorithm over a localized occupancy grid costmap. For online reactive control, both physical and virtual platforms deploy the dwb_core::DWBLocalPlanner controller, which samples candidate velocity vectors (v,ω) within non-holonomic acceleration bounds and minimizes a multi-objective cost function:
  (11) $$J\left(v,\omega \right)=w_{\mathrm{path}}\cdot C_{\mathrm{path}}\left(v,\omega \right)+w_{\mathrm{goal}}\cdot C_{\mathrm{goal}}\left(v,\omega \right)+w_{\mathrm{obs}}\cdot C_{\mathrm{obs}}\left(v,\omega \right)$$
  where $w_{i}$ represent tuned critic weights and $C_{i}\left(v,\omega \right)$ denote spatial alignment and obstacle proximity penalties.

This multi-tiered control architecture maintains matched high-level velocity commands and controller logic across domains while employing domain-specific odometry parameters: directly measured and documented runtime parameters for the physical UGV, and simulator-specific effective parameters $\left(r_{\mathrm{sim}},B_{\mathrm{sim}}\right)$ for the Isaac Sim digital twin.

#### 2.5.2. Multi-Trajectory Experimental Regimen and Trial Distributions

To prevent single-run anomalies from biasing the validation results, the experimental protocol mandates a distribution of ten physical trials (n=10) and ten simulated digital twin trials (n=10) for each designated path profile. Performance is evaluated across three distinct trajectory behaviors:

- Square Path Test (1.0m×1.0m): A 4.0m total perimeter profile designed to evaluate discrete $90^{\circ }$ turn maneuvers, isolating transient tracking errors, actuator deadbands, terminal closure errors, and orientation drift.
- Figure-8 Path Test: A continuous double-loop profile featuring a nominal radius R=0.50m per loop, corresponding to an approximately D=1.00m nominal loop diameter. This test evaluates continuous yaw tracking, direction-dependent turning asymmetry, and non-systematic odometry drift under alternating rotational motion. For the physical figure-eight trials, the endpoint was measured independently of the onboard EKF. Before each run, the robot was returned to the same marked initial position and orientation. The planar reference point was defined as the midpoint of the drive axle between the left and right wheels. After completing the maneuver, the final longitudinal and lateral floor offsets, Δx and Δy, of this reference point relative to the marked initial origin were measured manually. The physical closure displacement was then computed as $e_{c}=\sqrt{\Delta x^{2}+\Delta y^{2}}$. These measurements provide an external endpoint-displacement reference independent of /odometry/filtered, although they do not constitute motion-capture-grade ground truth.
- Maze Navigation Course: An integrated indoor environment evaluating SLAM-based mapping, global path planning, local costmap reactivity, and dynamic obstacle avoidance.

Beyond the nominal repeated physical–virtual trials, Stage 3 was further evaluated through dedicated local-sensitivity and robustness/generalization campaigns comprising 15 repeated trials per controlled condition, as detailed in Section 2.5.6 and Section 2.6.

#### 2.5.3. Temporal and Spatial Synchronization Protocol

Cross-domain trajectory tracking requires temporal and spatial alignment to support consistent metric comparison between physical and simulated trials.

- Temporal Alignment: Virtual trials inside Isaac Sim utilize the simulator’s internal clock topic (/clock) with the middleware parameter use_sim_time:=true. Physical trials record data locally using the system clock of the Jetson Orin Nano edge computer. The timelines for both domains are synchronized by setting t=0s at the first recorded timestamp where the commanded linear velocity exceeds the threshold v>0.01m/s.
- Spatial Alignment: Let $p_{k}={\left[x_{k},y_{k}\right]}^{⊤}$ represent the planar coordinate location of the UGV base frame at index *k*. Because external motion-capture tracking hardware was not available, physical coordinates were derived from the available onboard state-estimation source for each stage: EKF-filtered odometry for low-level trajectory trials and AMCL pose estimates in the SLAM-derived map frame for navigation trials. These estimates are treated as physical reference estimates, not as absolute ground truth. The trajectories from both domains are anchored to a common origin context at the initiation of motion:
  (12) $$p_{\mathrm{sim},0}=p_{\mathrm{real},0}={\left[0,0\right]}^{⊤},\;\theta_{\mathrm{sim},0}=\theta_{\mathrm{real},0}=0.$$

#### 2.5.4. Cross-Domain Trajectory Deviation Metrics

Because physical tracking relies on onboard state-estimation sources rather than independent external tracking instrumentation, the tracking results are explicitly defined as cross-domain deviations rather than absolute tracking errors. Depending on the stage, the physical reference estimate is derived from EKF-filtered odometry, manual floor measurements, or AMCL pose estimates in the SLAM-derived map frame. The tracking divergence across *K* synchronized path states is quantified through three statistical metrics:

- Cross-Domain Translational Trajectory Deviation ($D_{\mathrm{trans}}$): Measures the root-mean-square spatial discrepancy between the simulator reference trajectory and the corresponding physical reference estimate available for that stage.
  (13) $$D_{\mathrm{trans}}=\sqrt{\frac{1}{K}\sum_{k=1}^{K}{∥p_{\mathrm{sim},k}-p_{\mathrm{real},k}∥}^{2}}$$
- Cross-Domain Rotational Discrepancy ($D_{\mathrm{rot}}$): Quantifies cumulative heading drift errors across the execution run.
  (14) $$D_{\mathrm{rot}}=\sqrt{\frac{1}{K}\sum_{k=1}^{K}{\left(\theta_{\mathrm{sim},k}-\theta_{\mathrm{real},k}\right)}^{2}}$$
- Map Structural Similarity Index Measure (SSIM): To evaluate mapping consistency at the end of the maze navigation course, the physical occupancy grid map ($M_{\mathrm{real}}$) and the virtual layout map ($M_{\mathrm{sim}}$) are rasterized to matching 8-bit grayscale layouts and compared using structural similarity tracking:
  (15) $$\mathrm{SSIM}\left(M_{\mathrm{sim}},M_{\mathrm{real}}\right)=\frac{\left(2\mu_{\mathrm{sim}}\mu_{\mathrm{real}}+C_{1}\right)\left(2\sigma_{\mathrm{sim},\;\mathrm{real}}+C_{2}\right)}{\left(\mu_{\mathrm{sim}}^{2}+\mu_{\mathrm{real}}^{2}+C_{1}\right)\left(\sigma_{\mathrm{sim}}^{2}+\sigma_{\mathrm{real}}^{2}+C_{2}\right)}$$
  where $\mu_{i}$ and $\sigma_{i}^{2}$ are the spatial mean and variance parameters of the grids, and $C_{1}$, $C_{2}$ are stabilization constants.

#### 2.5.5. Statistical Reporting and Safety Evaluation Framework

Performance was evaluated over the complete multi-run trial distributions rather than through single-run observations or unsupported idealized obstacle-avoidance claims. Target values were treated as engineering design objectives and not as rigid acceptance thresholds. The statistical summary was selected according to the distributional characteristics of each response variable. For continuous Stage 3 outcomes, including baseline-referenced trajectory deviation, minimum obstacle clearance, and navigation time, results are reported primarily as median values with interquartile ranges (IQRs), because several perturbation conditions produced skewed or multimodal distributions. Task-completion rates were calculated from all attempted trials, including unsuccessful or aborted runs, rather than only from completed runs.

Safety-related navigation behavior was summarized using the following performance vector:

(16) $$S_{\mathrm{vector}}=\left[\begin{array}{ccccc} P_{\mathrm{success}} & C_{\mathrm{count}} & d_{\min} & E_{\mathrm{recovery}} & t_{\mathrm{nav}} \end{array}\right]$$

where $P_{\mathrm{success}}$ is the proportion of successful goal completions across all attempted trials, $C_{\mathrm{count}}$ is the recorded number of physical contact or collision events, $d_{\min}$ is the minimum geometric clearance from the obstacle, $E_{\mathrm{recovery}}$ represents the recovery actions invoked by the navigation stack, and $t_{\mathrm{nav}}$ is the navigation time measured from the NavigateToPose feedback using simulation time for virtual trials. The vector was interpreted together with the complete distribution of trial outcomes, allowing repeatability, sensitivity to controlled perturbations, and the effects of recovery behavior to be assessed without discarding anomalous or unsuccessful runs.

#### 2.5.6. Stage 3 Local Parameter Sensitivity Analysis

A one-factor-at-a-time (OFAT) local sensitivity analysis was conducted around the active Stage 3 closed-loop navigation configuration to quantify the dependence of digital-twin behavior on effective wheel geometry and PhysX joint-drive damping. The verified baseline parameters were an effective wheel radius of $r_{0}=0.03300\;m$, wheel separation of $B_{0}=0.19800\;m$, and PhysX angular-drive damping of $D_{0}=10,000$. Six perturbed conditions were evaluated: r±5%, B±5%, and D±10%. Fifteen independent navigation trials were conducted for each condition and for the unperturbed baseline, yielding 105 runs in total.

(17) $$p=p_{0}\left(1+\delta_{p}\right),\;p\in \left\{r,B,D\right\},$$

with

(18) $$\delta_{r},\delta_{B}\in \left\{-0.05,+0.05\right\},\;\delta_{D}\in \left\{-0.10,+0.10\right\}.$$

The sensitivity campaign was defined around the active Stage 3 differential-controller and wheel-odometry configuration. The corresponding operating-point parameters were distinct from the effective virtual kinematic parameters used in the earlier V16/V17 calibration models. In each condition, only one of the effective parameters was varied, while all remaining navigation, obstacle, map, planner, and sensor settings were held fixed. The perturbations modified the effective wheel-odometry and controller geometry; the physical wheel collision meshes themselves were not geometrically resized. The perturbation amplitudes were selected as controlled local sensitivity bands around the verified operating point and are therefore not interpreted as experimentally estimated confidence intervals or as direct measurements of calibration uncertainty.

All non-tested navigation parameters were held constant, including the DWB local planner, robot radius (0.16m), inflation radius (0.25m), cost-scaling factor (10.0), minimum angular velocity ($0.30\;\mathrm{rad}\;s^{-1}$), goal tolerances, start and goal poses, dynamic-cylinder geometry, and the nominal 0.120m ground-truth displacement used to trigger obstacle insertion. Runtime wheel-odometry parameters and PhysX joint-drive values were recorded and verified for every condition (see Table 4).

Three primary response variables were evaluated. Baseline-referenced trajectory deviation was computed from Isaac Sim rigid-body ground truth as the arc-length-weighted root-mean-square nearest-point distance between each initial-pose-aligned trajectory and the median calibrated C00 baseline trajectory. For C00, leave-one-out baseline references were used to quantify intrinsic run-to-run repeatability.

Minimum obstacle clearance was defined as the minimum post-insertion planar geometric separation between the dynamic cylinder and the conservative robot collision envelope extracted from the Stage 3 USD model. This metric is therefore interpreted as a conservative geometric clearance rather than as a high-fidelity mesh-to-mesh contact distance.

Baseline-referenced trajectory deviation was computed for each run using the arc-length-weighted RMS nearest-point distance to the median calibrated baseline trajectory:

(19) $$D_{\mathrm{traj},i}=\sqrt{\frac{\sum_{k=1}^{K_{i}}w_{i,k}\;d^{2}\;\left(p_{i,k},P_{0}\right)}{\sum_{k=1}^{K_{i}}w_{i,k}}},$$

where the nearest-point distance is defined as

(20) $$d\;\left(p,P_{0}\right)=\min_{q\in P_{0}}{∥p-q∥}_{2}.$$

Here, $p_{i,k}$ denotes the initial-pose-aligned /ground_truth/odom point at sample *k* of run *i*, $P_{0}$ denotes the median ground-truth polyline of the calibrated C00 baseline, and $w_{i,k}$ is the local arc-length weighting. For the C00 runs, leave-one-out baseline references were used so that the baseline value represents intrinsic run-to-run trajectory repeatability rather than a trivial zero-distance comparison.

Minimum obstacle clearance was defined as the minimum geometric separation between the robot collision envelope and the dynamic cylinder after obstacle insertion:

(21) $$d_{\min}=\min_{t\geq t_{\mathrm{ins}}}d_{\mathrm{geom}}\left(B_{\mathrm{robot}}\left(t\right),C_{\mathrm{cyl}}\right),$$

where $t_{\mathrm{ins}}$ denotes the dynamic-cylinder insertion time, $B_{\mathrm{robot}}\left(t\right)$ is the conservative planar oriented collision envelope of the robot, and $C_{\mathrm{cyl}}$ is the cylindrical obstacle with radius 0.125m. This quantity is defined as a conservative geometric clearance, not an exact high-fidelity mesh-to-mesh distance. The conservative collision-envelope bounds used for the Stage 3 clearance calculation are documented in Appendix D.3.

As defined in Equation (19), the trajectory deviation was calculated relative to the median calibrated C00 baseline polyline. The distance function used in Equation (19) is defined in Equation (20). Minimum obstacle clearance was calculated according to Equation (21).

Navigation time was obtained from the maximum NavigateToPose feedback value for each run, using simulation time rather than rosbag wall-clock duration. Because several perturbation conditions produced skewed or multimodal distributions, continuous outcomes are reported as median and interquartile range (IQR). Continuous metrics were calculated from completed runs, whereas all attempted runs contributed to the task-completion rate.

### 2.6. Stage 3 Robustness and Generalization Protocol

A dedicated virtual Stage 3 robustness and generalization campaign evaluated five controlled conditions with 15 independent navigation trials per condition, yielding 75 trials in total. C00 was the calibrated nominal baseline; G01 shifted the dynamic cylindrical obstacle by +0.15m along the *Y* axis; G02 shifted it by −0.15m; G03 applied a low-friction surface with $\mu_{s}=0.30$ and $\mu_{d}=0.25$; and G04 introduced LiDAR degradation consisting of an additional Gaussian range-noise standard deviation of 0.03m together with 5% random beam dropout. All non-tested navigation, map, obstacle, planner, and robot-model parameters were held constant across conditions.

## 3. Experimental Results

### 3.1. Experimental Performance Metrics and Reference-Pose Framework

To establish methodological rigor across physical and virtual domains without making unverified localization claims, this work distinguishes between simulator-provided reference poses, physical state-estimation outputs, and cross-domain trajectory deviations.

- Virtual-domain reference pose (${\mathrm{GT}}_{\mathrm{sim}}$): In NVIDIA Isaac Sim 4.5.0, the simulator provides a rigid-body reference pose through the /ground_truth/odom topic. This signal is not used by the controller and is used only for offline evaluation of virtual estimator behavior, endpoint closure, cross-track deviation, and yaw discrepancy. Since this reference is generated by the simulator, it is treated as simulator ground truth, not as physical-world ground truth.
- Physical-domain state estimation (${\mathrm{SE}}_{\mathrm{phys}}$): In the physical UGV trials, no external motion-capture, total-station, or overhead tracking system was deployed. Physical trajectories are therefore derived from onboard state-estimation sources, including EKF-filtered odometry /odometry/filtered and, in navigation experiments, AMCL pose tracking. Consequently, physical trajectory metrics quantify closed-loop repeatability, estimator consistency, endpoint displacement, and navigation behavior rather than absolute localization error with respect to an external ground-truth system.
- Cross-domain trajectory deviation ($\Delta_{\mathrm{cross}}$): Differences between physical execution and digital-twin execution are reported as cross-domain trajectory deviations rather than absolute trajectory errors. These deviations quantify the Real-to-Sim behavioral gap under matched commands, reference trajectories, map-frame conventions, controller settings, and obstacle configurations. Accordingly, terms such as translational deviation, rotational discrepancy, cross-track deviation, endpoint closure, and map-similarity metrics are preferred over unqualified “absolute trajectory error” when physical ground truth is unavailable.

### 3.2. Preliminary Physical Trajectory Verification via EKF-Assisted Control

To evaluate the operational repeatability and drift characteristics of the low-level embedded kinematics prior to full exteroceptive SLAM integration, a multi-run experimental regimen was conducted on the physical UGV chassis. The platform executed a closed-loop 1.0m×1.0m nominal square trajectory over ten consecutive repeated trials (n=10). Two state-evaluation paradigms were logged concurrently: raw uncorrected wheel odometry and the asynchronous multi-rate extended Kalman filter (EKF) output fusing encoder-derived wheel-odometry velocities with IMU yaw-rate measurements. The consolidated statistical distributions derived from the ten physical runs are detailed in Table 5.

**Figure 5:**
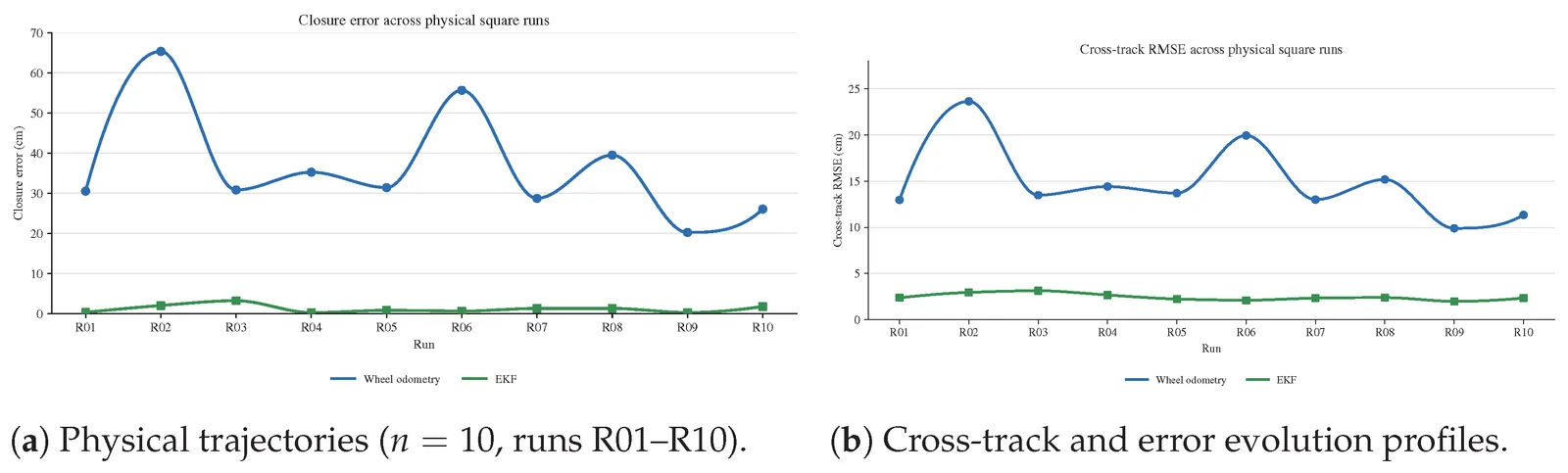
Experimental results for the physical 1.0m×1.0m square-path validation across ten repeated runs: (**a**) per-run closure error for raw wheel odometry and EKF-assisted estimation; (**b**) corresponding per-run cross-track RMSE comparison.

The empirical evaluation shows substantial reductions in the reported error metrics when EKF state feedback is used. The onboard-estimated mean trajectory closure error decreased from 36.35±13.90cm with raw wheel odometry to 1.18±0.96cm with EKF-assisted feedback, corresponding to a 96.8% reduction. Incorporating the MPU-6050 yaw-rate measurement reduced the mean absolute final heading deviation from 25.97±10.21° to 1.41±1.02°. The geometric RMSE decreased by 83.3%, from 14.75±4.09cm to 2.46±0.36cm under fused guidance, as summarized in Figure 5.

### 3.3. Continuous Figure-Eight Physical Benchmarking and Sensor Fusion Analysis

To evaluate the platform’s kinematic response under continuous, alternating non-holonomic curvilinear motion without discrete deceleration segments, a continuous figure-eight validation sequence (N=10) was executed. The target profile comprised one counter-clockwise (CCW) loop followed continuously by one clockwise (CW) loop (R=0.50m nominal radius, 100cm nominal loop diameter). The aggregate statistical results across the ten independent physical trials (designated R01–R10) are detailed in Table 6.

The nominal geometric length of two circular loops with R=0.50m is 4πR=6.283m. However, the physical vehicle did not execute exactly 1.00m-diameter loops. The independently measured mean diameters were 1.0795m for the counter-clockwise loop and 1.1280m for the clockwise loop. Using these measured diameters gives an approximate geometric path length of π(1.0795+1.1280)=6.935m, which is consistent with the EKF-reconstructed mean path length of 6.96m. Therefore, the apparent excess relative to the nominal 6.283m path results primarily from the larger physical turning radii rather than from EKF path-length accumulation alone.

**Figure 6:**
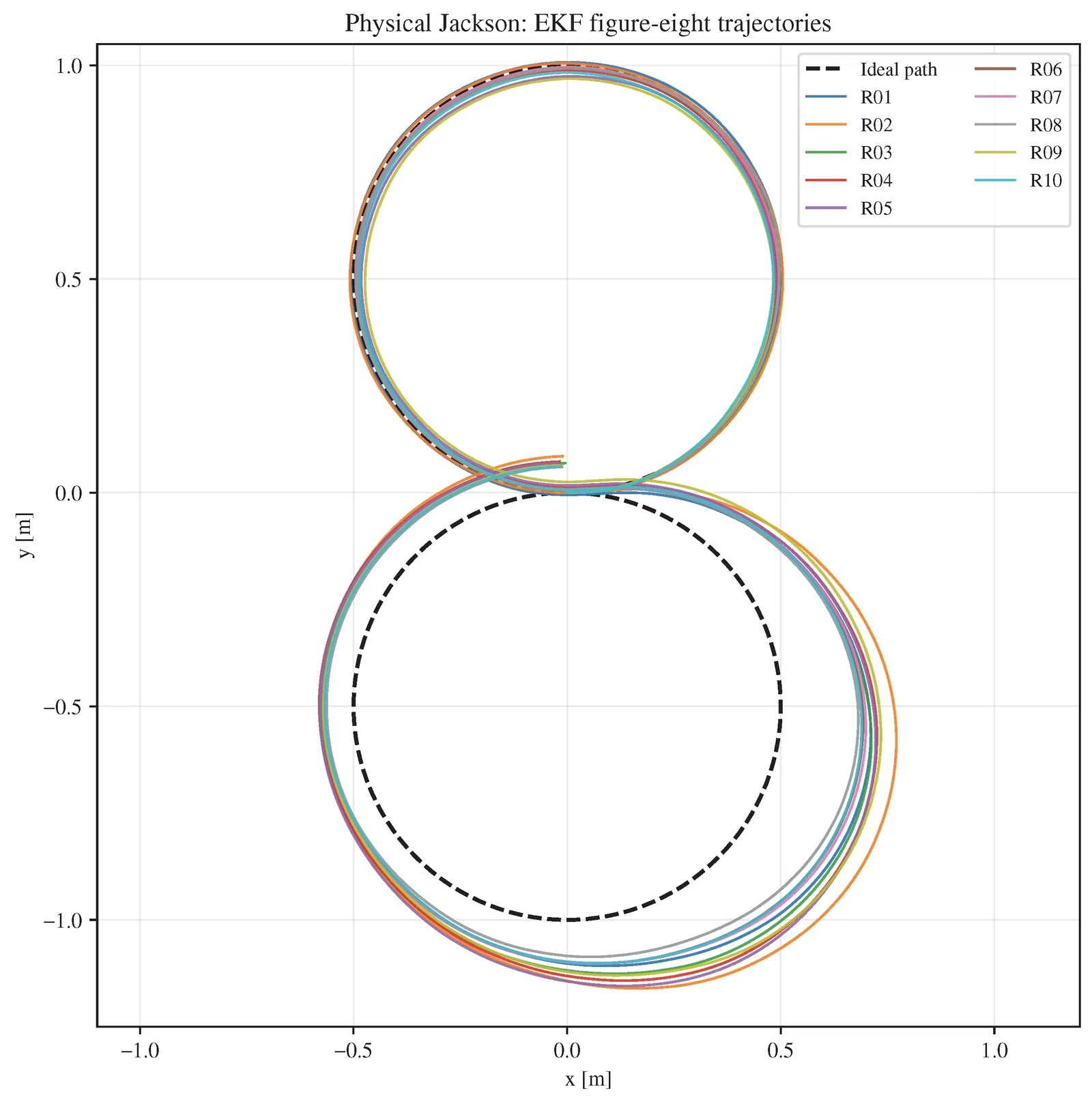
Reconstructed spatial EKF trajectories across ten repeated physical figure-eight trials (N=10) overlaid against the nominal R=0.50m reference path.

**Figure 7:**
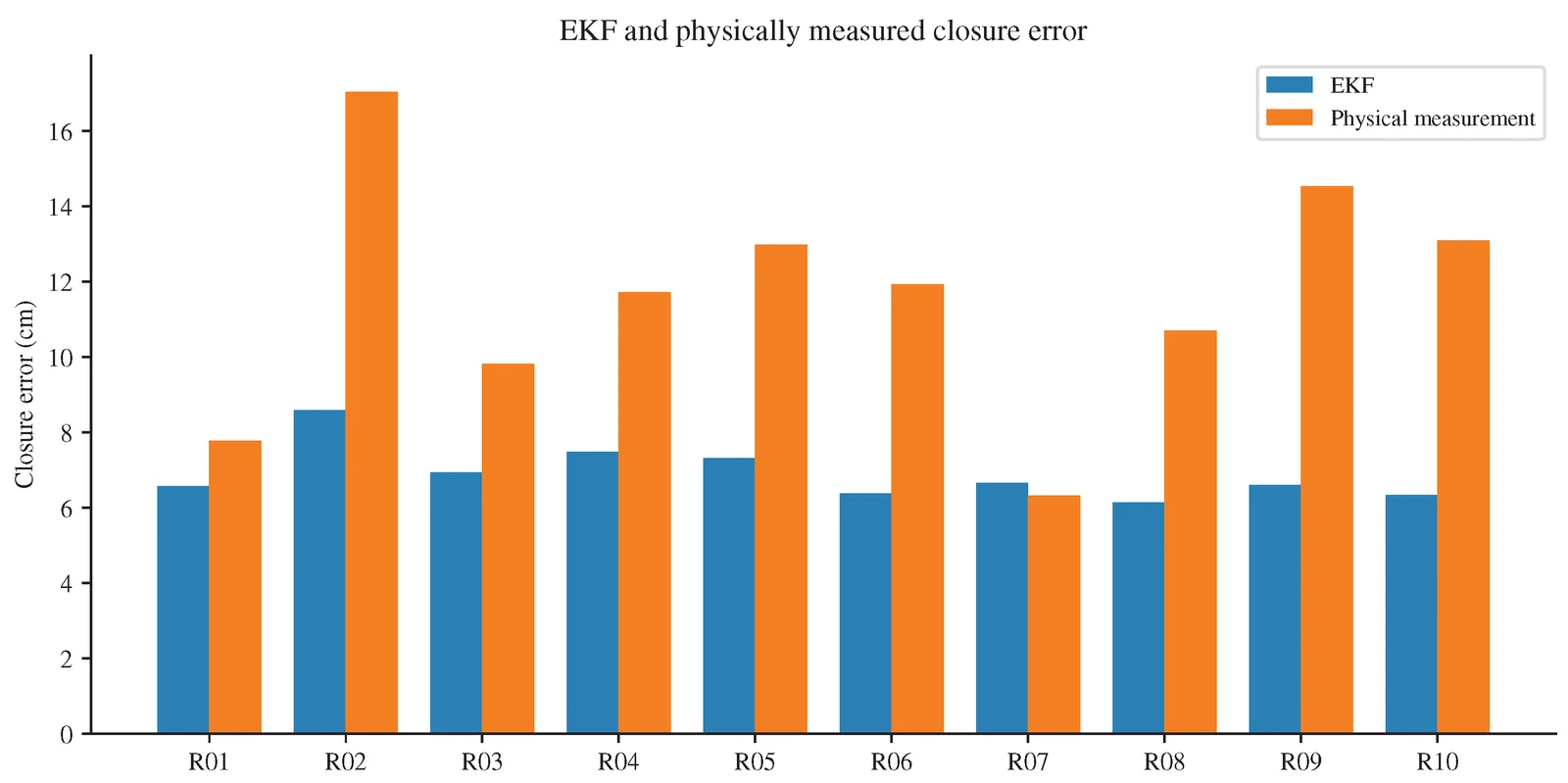
Spatial endpoint dispersion and physical vs. estimator closure error after completing the continuous figure-eight maneuver.

**Figure 8:**
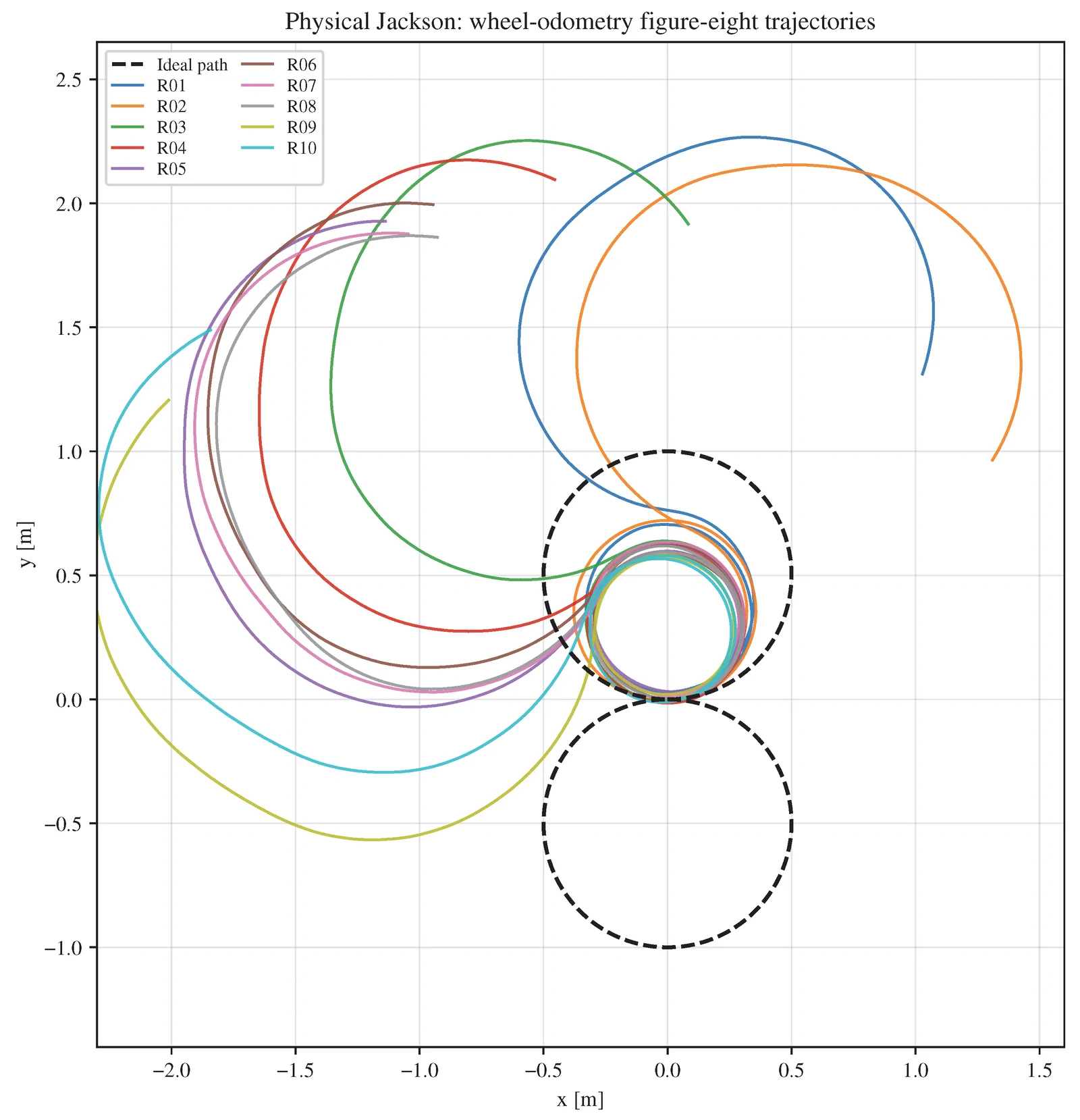
Diagnostic comparison highlighting severe unbounded yaw accumulation in unassisted wheel odometry (207.00±26.00cm error) during continuous alternating rotations.

The physical trials demonstrated a direction-dependent systematic geometric bias between clockwise and counter-clockwise maneuvers (107.95±1.67cm CCW vs. 112.80±3.22cm CW). As illustrated in Figure 6, all ten trajectories consistently reconstruct the continuous figure-eight topology. Figure 7 details the final posture dispersion, highlighting the systematic offset between estimated (6.90±0.73cm) and physically measured (11.59±3.14cm) closure coordinates. Figure 8 and Table 7 confirm that unassisted wheel odometry accumulates severe unbounded drift (207.00±26.00cm closure error), demonstrating the substantial benefit of EKF–IMU rate integration for this maneuver.

### 3.4. Digital Twin Trajectory Verification in NVIDIA Isaac Sim

To establish the virtual baseline, N=10 closed-loop 1.0m×1.0m square trajectory trials (R01–R10) were executed within NVIDIA Isaac Sim 4.5.0 using the V16 calibrated asset ($r_{\mathrm{eff}}=0.031650\;m$, $B_{\mathrm{eff}}=0.210481\;m$, IMU yaw scale 0.6953). Statistical performance metrics are summarized in Table 8.

**Figure 9:**
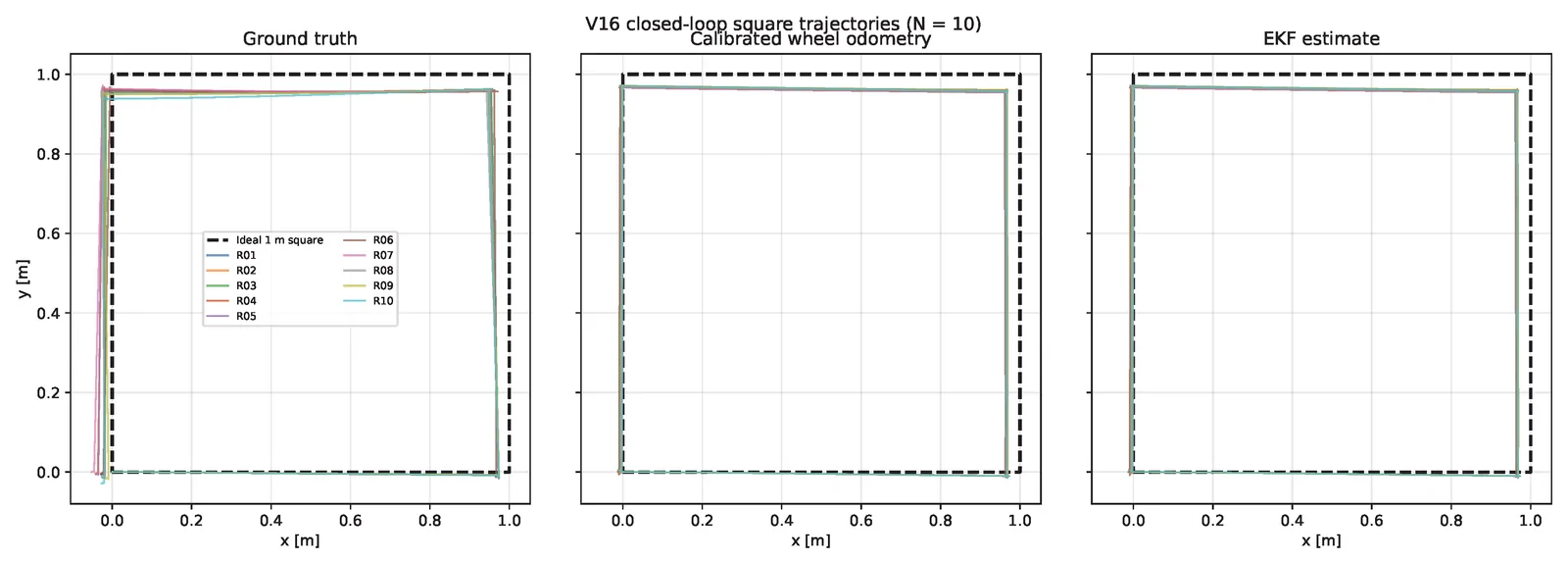
Simulated closed-loop 1.0m×1.0m square trajectories across ten independent runs (N=10) in Isaac Sim 4.5.0, comparing exact PhysX pose (**left**), calibrated wheel odometry (**center**), and ROS 2 EKF state estimation (**right**).

**Figure 10:**
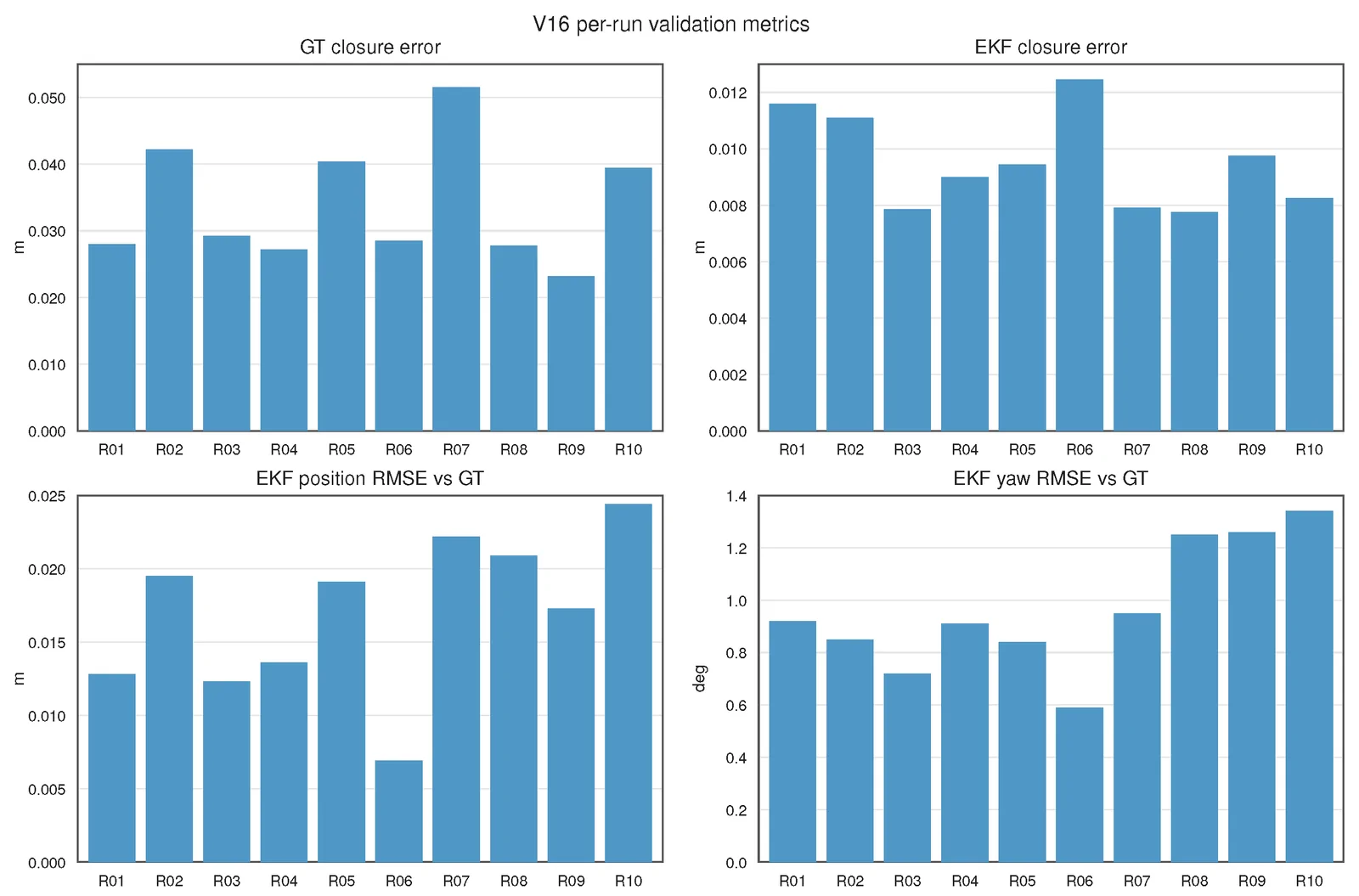
Per-run validation metrics across ten simulated trials (R01–R10), showing true PhysX closure error, EKF-estimated closure error, EKF position RMSE, and EKF yaw RMSE relative to the exact PhysX pose baseline.

Figure 9 displays the spatial trajectory overlays across PhysX reference poses, wheel odometry, and EKF state estimates, while Figure 10 details per-run performance. Evaluating internal filter states against the zero-latency rigid-body pose baseline reveals an inherent −2.43cm estimator optimism bias (0.95±0.17cm filter closure vs. 3.39±0.90cm exact PhysX pose).

**Figure 11:**
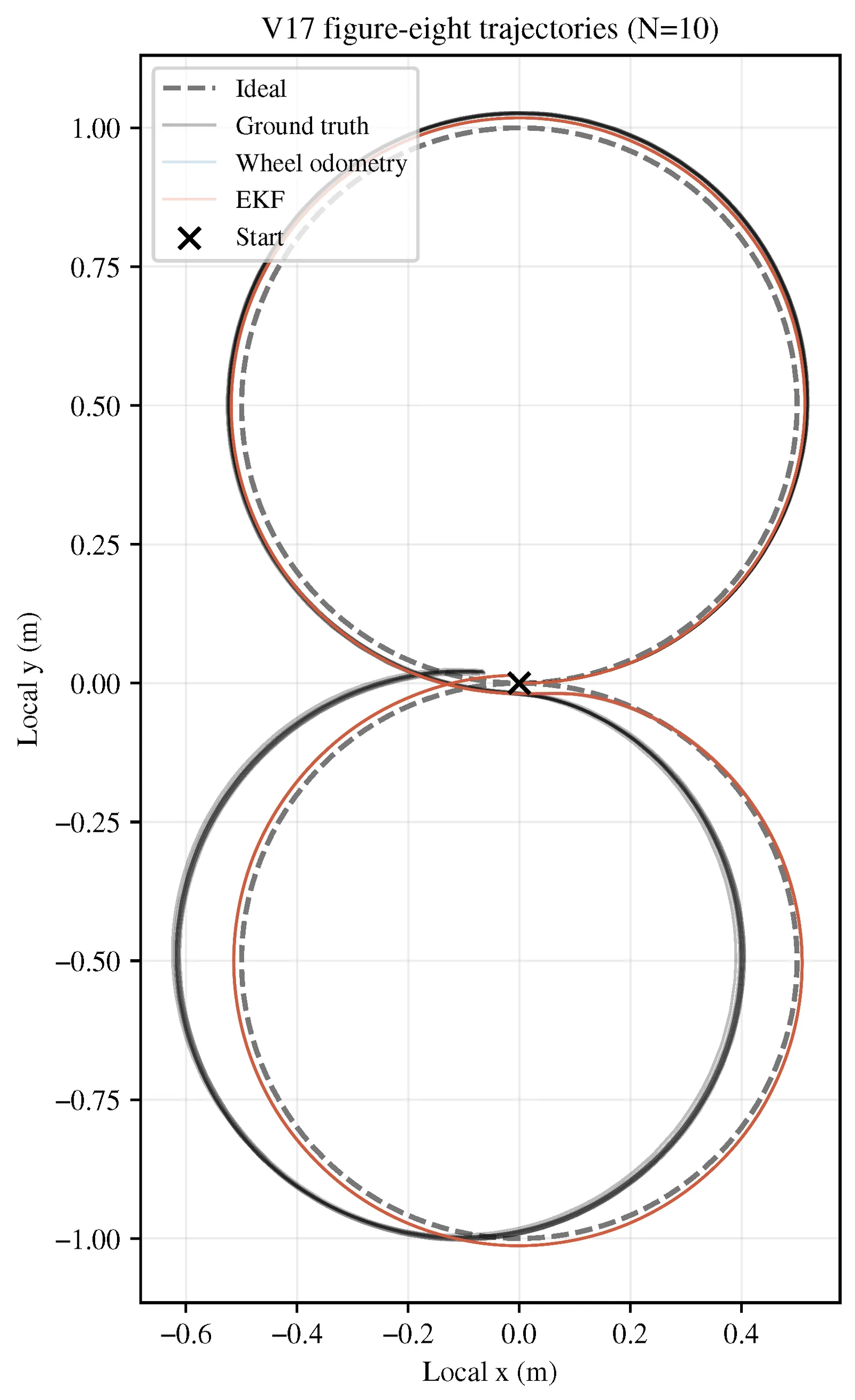
Reconstructed spatial trajectories across ten repeated virtual figure-eight trials (N=10) in NVIDIA Isaac Sim 4.5.0, showing systematic spatial displacement between the exact PhysX rigid-body pose and internal EKF/wheel odometry estimates.

Additionally, N=10 Stage 2 virtual figure-eight trials (R=0.50m) were executed within Isaac Sim 4.5.0. As shown in Figure 11, spatial tracking along the upper CCW loop aligns closely with reference geometry, while continuous rotational vector shifts in the lower CW loop induce a systematic spatial displacement in the exact PhysX reference state ($y_{\min}\approx -1.00\;m$) that wheel odometry and EKF estimates cannot observe.

**Figure 12:**
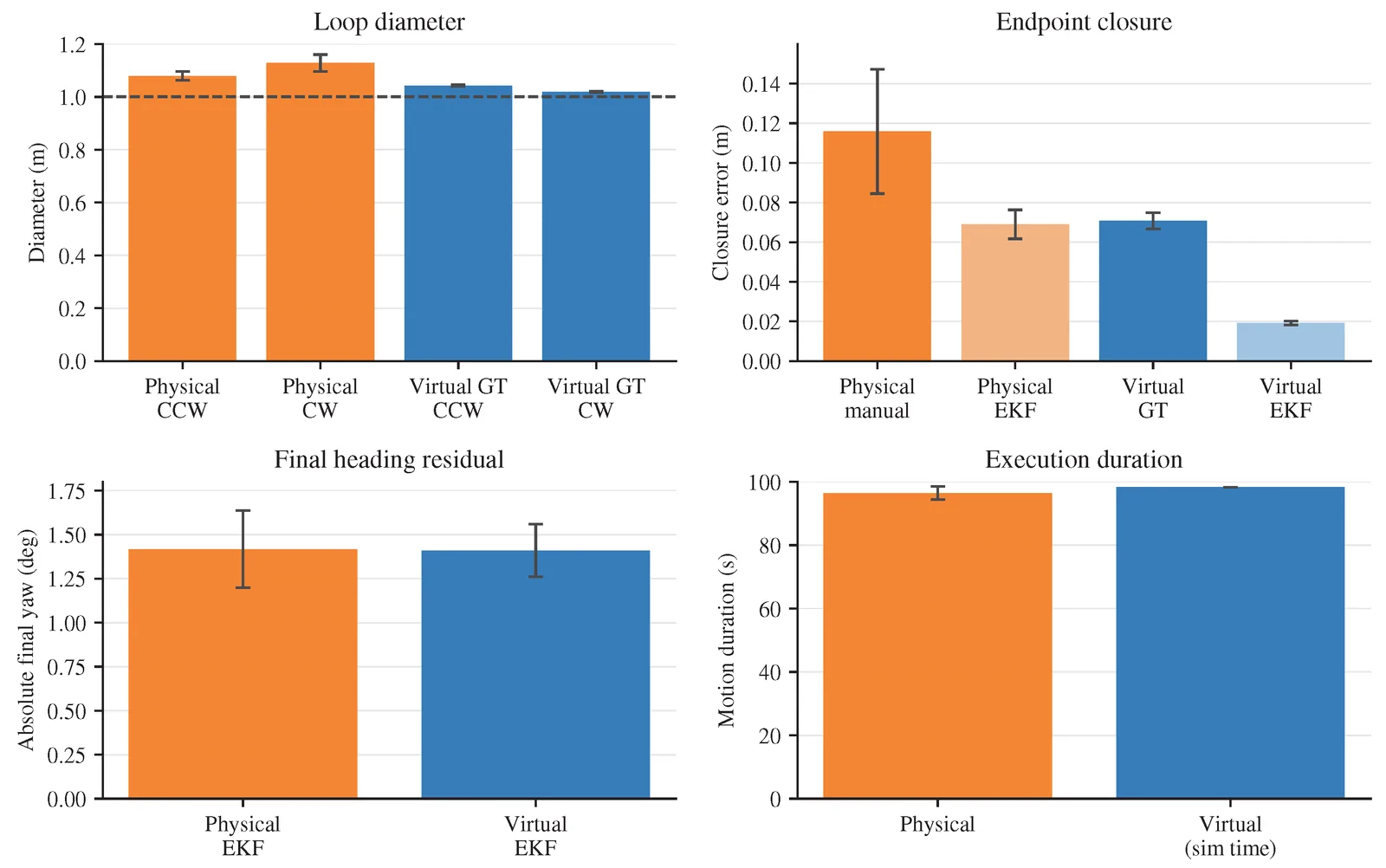
Direct quantitative comparison between physical UGV execution (N=10) and calibrated Isaac Sim digital twin simulation (N=10) for the Stage 2 continuous figure-eight trajectory across four key performance metrics: loop diameter asymmetry, endpoint closure error (highlighting double-domain estimator optimism), absolute final heading residual, and total motion duration.

The corresponding cross-domain comparison of loop geometry, endpoint closure, final heading residual, and motion duration is summarized in Figure 12.

### 3.5. Integrated Maze Navigation, SLAM, and Dynamic Obstacle Avoidance

All coordinate locations in Stage 3 are expressed in the ROS 2 map frame associated with the 2D occupancy grid generated during initial physical mapping via slam_toolbox. The physical occupancy-grid frame serves as the common spatial reference for both the real robot and the NVIDIA Isaac Sim digital twin reconstruction.

The platform was commanded to traverse a 1.900m nominal straight-line route from initial pose $\left(x_{0},y_{0},\theta_{0}\right)=\left(-0.6909\;m,-0.0364\;m,0.0\;\mathrm{rad}\right)$ to target goal pose $\left(x_{g},y_{g},\theta_{g}\right)=\left(1.2091\;m,-0.0364\;m,0.0\;\mathrm{rad}\right)$, yielding a net route displacement of $x_{g}-x_{0}=1.900\;m$.

To evaluate online dynamic obstacle avoidance across both domains, a rigid cylindrical obstacle ($r_{c}=0.125\;m$ radius) was dynamically inserted into the transit corridor after motion execution had commenced. The obstacle center was positioned 1.300m ahead of the start pose along the map-frame *x*-axis, corresponding to coordinate location $\left(x_{c},y_{c}\right)=\left(x_{0}+1.300\;m,y_{0}\right)=\left(0.6091\;m,-0.0364\;m\right)$ (approximately 68.4% of the start-to-goal path length). The insertion was triggered at 0.1217±0.0012m of forward simulation displacement, ensuring the obstacle was absent from the initial static costmap at motion onset and forcing real-time local costmap re-planning without global path pre-computation.

Local navigation across both physical and virtual domains was governed by the dwb_core::DWBLocalPlanner controller with a 0.16m footprint radius, 0.25m inflation radius, and 0.25m XY goal tolerance. Both physical (100%, 10/10) and virtual (100%, 10/10) trials achieved full task completion ($P_{\mathrm{success}}=1.0$) with no observed obstacle-contact events ($C_{\mathrm{count}}=0$).

**Figure 13:**
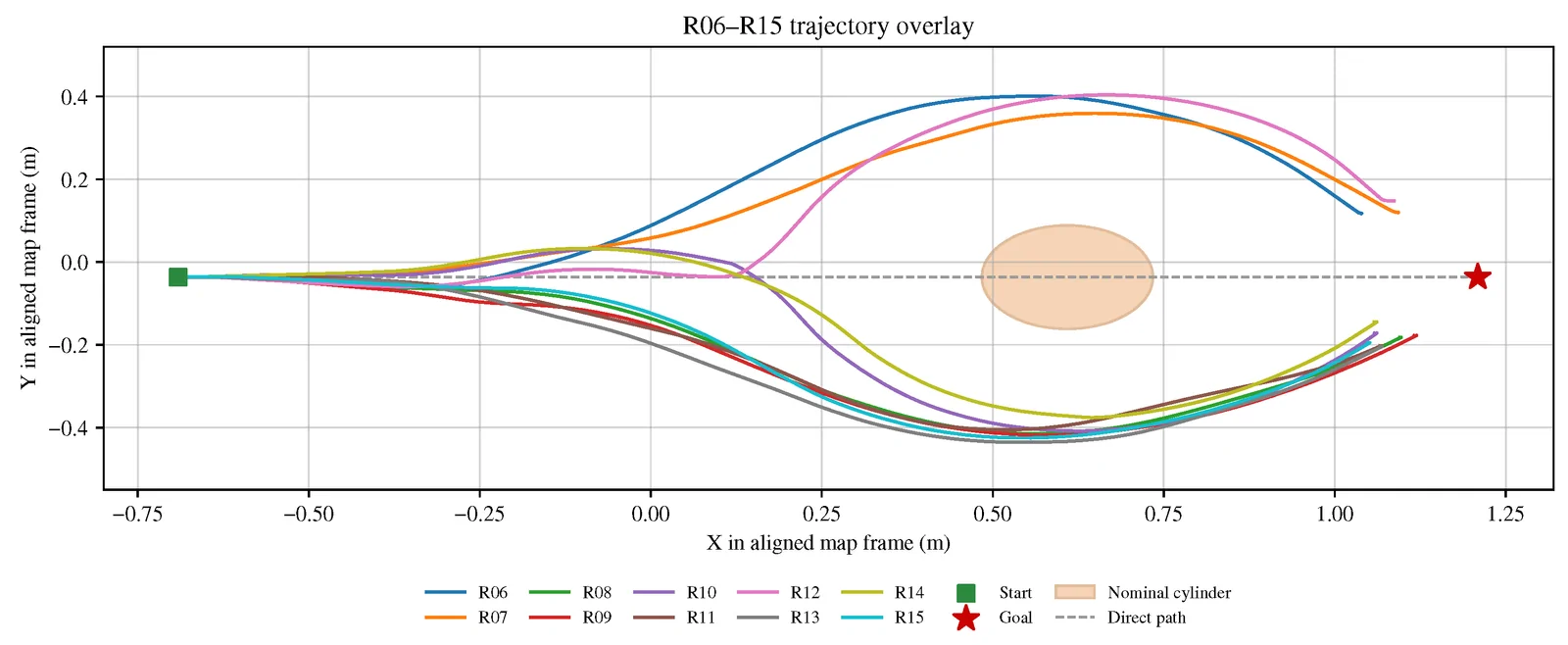
Reconstructed spatial trajectories obtained from the fused /odometry/filtered pipeline across the official physical Maze Navigation dataset (n=10, runs R06–R15). A cylindrical obstacle (12.5cm radius) was dynamically inserted into the corridor at (0.6091,−0.0364m) after motion initiation along the 1.900m straight-line path. The Nav2 local costmap dynamically re-routed the local trajectory, preserving a safe minimum obstacle clearance ($d_{\min}=0.122\pm 0.007\;m$) with 100% task success ($P_{\mathrm{success}}=1.0$) and zero collisions ($C_{\mathrm{count}}=0$).

**Figure 14:**
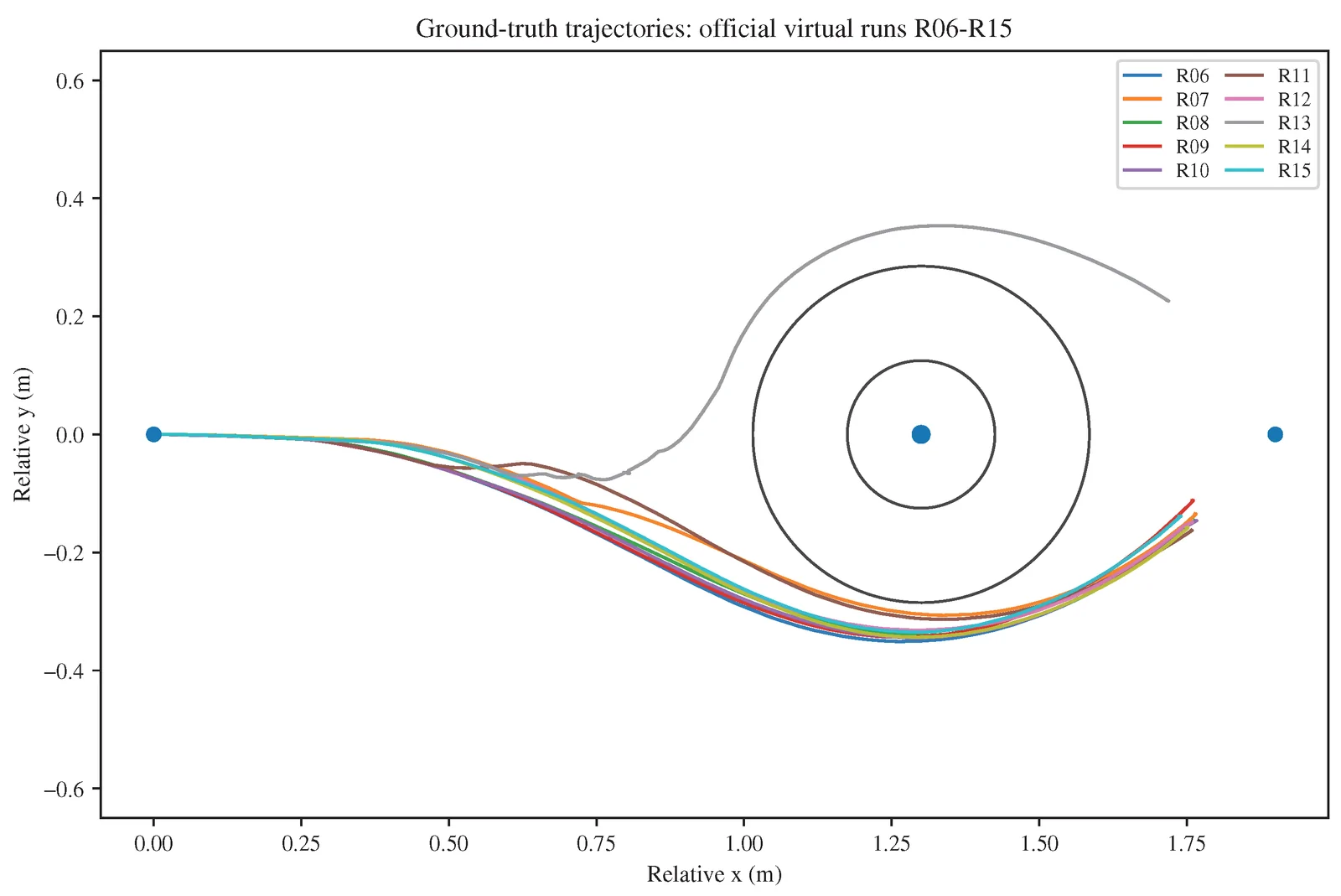
Ground-truth spatial trajectories obtained across the official virtual Stage 3 dynamic obstacle runs (N=10, R06–R15) in NVIDIA Isaac Sim. The dynamic cylinder (0.125m radius) was triggered at 0.1217±0.0012m of forward displacement at $x_{c}=0.6091\;m$. The blue markers indicate the initial robot position, the center of the dynamic cylindrical obstacle, and the navigation goal, respectively. Nine runs selected the lower route, while run R13 represents a valid operational outlier selecting the upper route after 25 global plan-side switches and 5 recovery actions. All runs completed with 100% success without geometric intersection with the cylindrical obstacle ($d_{\min}=0.074\pm 0.016\;m$).

Figure 13 shows the physical spatial trajectories generated by the Jackson UGV, while Figure 14 presents the corresponding ground-truth virtual trajectories in Isaac Sim. Nine of the ten virtual runs (R06–R12, R14–R15) selected the lower avoidance route around the cylinder. Run R13 represents an authentic operational outlier: while completing successfully without contact (60.150s execution duration, 1.994m path length), local costmap oscillation triggered 25 global plan-side switches and 5 recovery maneuvers before the planner safely executed the upper route.

### 3.6. Stage 3 Local Parameter Sensitivity Results

The results in Table 9 establish the calibrated C00 condition as a repeatable reference, with an intrinsic median baseline-referenced trajectory deviation of 1.05 cm [0.98, 2.22 cm]. The strongest systematic geometric effect was produced by increasing the effective wheel radius by 5%, which increased the median trajectory deviation from 1.05 to 35.80 cm, reduced the median obstacle clearance from 8.17 to 4.95 cm, and increased the median navigation time from 13.23 to 25.20 s, corresponding to a 90.4% increase. In contrast, reducing the effective wheel separation by 5% produced the strongest operational instability: the median navigation time increased to 54.31 s, median clearance decreased to 5.65 cm, and only 14 of 15 trials completed successfully. The unsuccessful trial was retained as an observed aborted outcome and was not manually repeated.

The response was asymmetric and direction-dependent, since equal positive and negative perturbations did not produce equivalent behavior. Although the r−5% condition yielded a relatively small median trajectory deviation of 2.24 cm, its IQR extended to 37.42 cm, and the corresponding runs selected both negative-*Y* and positive-*Y* obstacle-avoidance paths, indicating multimodal rather than uniformly shifted behavior. Within the tested ±10% range, PhysX damping was comparatively less influential: the D−10% and D+10% conditions produced median trajectory deviations of 1.29 and 1.17 cm and median navigation times of 13.90 and 13.30 s, respectively, although D−10% reduced the median clearance to 6.90 cm. These findings indicate that Stage 3 navigation was more sensitive to effective wheel geometry than to the tested damping perturbations, without implying that larger damping changes would have the same limited effect.

### 3.7. Stage 3 Robustness and Generalization Results

To further evaluate robustness and generalizability, an additional virtual Stage 3 campaign was conducted under controlled variations in obstacle placement, wheel–ground interaction, and exteroceptive sensing. Fifteen trials were performed for each condition, yielding 75 trials in total. As shown in Table 10, all conditions achieved 15/15 successful goal completions. No geometric intersection with the cylindrical obstacle was observed in any of the 75 trials; the conservative minimum clearance remained positive in every run. Moving the cylindrical obstacle laterally by ±0.15m changed the selected avoidance geometry and produced shorter paths and navigation times than the centered baseline. The low-friction condition remained close to the baseline response, with a median navigation time of 12.33s compared with 13.23s for the baseline. In contrast, the degraded-LiDAR condition produced the greatest increase in execution variability: the median navigation time increased to 18.12s, with an IQR of 13.23–32.87s, while the median path length increased from 1.885 to 1.917m. The LiDAR perturbation was independently verified from the recorded ROS 2 bags, yielding an actual beam-dropout rate of 4.997% and an additional range-noise standard deviation of 0.03001m. The longest degraded-LiDAR trials were also associated with repeated Nav2 recovery actions, indicating that perception degradation primarily increased planning and recovery effort rather than preventing task completion.

### 3.8. Cross-Domain Real-to-Sim Trajectory Benchmarking

To complement the trajectory-quality metrics with an explicit temporal assessment, Table 11 summarizes the motion/navigation duration observed in the physical and virtual domains together with the available simulator wall-clock runtime. This distinction is important because Isaac Sim simulation time represents the temporal evolution experienced by the virtual robot, whereas wall-clock runtime reflects the computational time required to execute the simulation on the host system.

The temporal results distinguish robot-motion performance from simulator computational execution. For Stage 1, the physical motion interval was 47.52±0.26 s, while the corresponding digital-twin motion required 67.17±0.15 s of Isaac Sim simulation time. The same virtual trials required 94.97±4.69 s of wall-clock execution, demonstrating that simulator computational runtime and simulated robot-motion duration are distinct quantities. Stage 2 exhibited closely matched motion durations between the physical and virtual domains, whereas Stage 3 showed greater virtual timing variability because navigation recovery and replanning behavior differed among runs. Separate wall-clock computational timings were not recorded for Stages 2 and 3 and are therefore not estimated retrospectively.

To evaluate cross-domain transferability, comparative matrices were compiled across Stage 1 (Square), Stage 2 (Figure-Eight), and Stage 3 (Maze/Dynamic Obstacle Avoidance) experimental regimens. Table 12 summarizes the Stage 1 square metrics, Table 13 and Figure 12 detail the Stage 2 continuous figure-eight evaluation, while Table 14 synthesizes Stage 3 exteroceptive navigation performance.

The Stage 1 virtual motion duration reported in Table 11 was measured using Isaac Sim simulation time and is therefore directly comparable with the physical motion interval. The corresponding wall-clock runtime reported in Table 8 was 94.97±4.69s and reflects simulator computational execution rather than robot-motion duration. Wall-clock runtime is therefore reported separately and is not used as the cross-domain motion-time metric.

The cross-domain evaluation shows close numerical agreement in the final heading residual (1.42±0.22° physical vs. 1.41±0.15° virtual, Δ=0.01°) and execution duration (96.43s physical vs. 98.26s virtual, Δ=1.83s) during continuous figure-eight turns. At the same time, the comparison quantifies a consistent proprioceptive “estimator-optimism gap” across both domains (4.69cm physical vs. 5.16cm virtual, Δ=0.47cm).

In Stage 3 dynamic obstacle avoidance, both physical (n=10) and virtual (N=10) platforms achieved 100% goal completion ($P_{\mathrm{success}}=1.0$) with zero collisions ($C_{\mathrm{count}}=0$). Terminal goal-error magnitudes were similar across domains and remained within the configured Nav2 goal-checker tolerance: 0.211±0.011m for the physical AMCL estimate and 0.212±0.030m for the virtual ground-truth pose (Δ=0.001m). This comparison indicates terminal navigation-behavior similarity, not absolute localization equivalence. However, domain-dependent execution differences emerged under the identical DWB local planner (dwb_core::DWBLocalPlanner): the virtual digital twin executed a tighter trajectory (1.888±0.039m mean path length) passing closer to the obstacle boundary ($d_{\min}=0.074\pm 0.016\;m$) compared to physical execution (1.958±0.014m path length, $d_{\min}=0.122\pm 0.007\;m$). Median virtual navigation duration (13.317s) was lower than physical motion duration (22.405s), reflecting conservative physical motor acceleration limits, command transport latency, and wheel-surface friction differences (detailed in Table 14).

Overall, the cross-domain benchmarking demonstrates that dead-reckoning state estimation exhibits a systemic optimism bias across both domains (∼4.7–5.2cm closure error). The calibrated digital twin reproduces the main qualitative navigation behavior while preserving measurable domain-dependent differences in path length, clearance, and duration.

## 4. Discussion

To avoid conflating metrics obtained from different experimental stages, the cross-domain findings are discussed separately for the three validation levels: square waypoint tracking repeatability (Stage 1), continuous figure-eight turning dynamics and estimator optimism (Stage 2), and closed-loop exteroceptive navigation with dynamic obstacle avoidance (Stage 3). This stage-specific organization also preserves the distinct reference sources and performance metrics associated with each experimental level.

### 4.1. Stage 1: Square Waypoint Tracking Repeatability and EKF Consistency

Stage 1 evaluated low-level motion-command execution and EKF consistency over a 1.0m×1.0m square trajectory. The purpose of this stage was to isolate discrete linear segments, approximately $90^{\circ }$ turns, endpoint closure, yaw convergence, and trajectory repeatability before introducing continuous turning or obstacle-avoidance behavior.

In the physical UGV experiments (n=10), the EKF-guided controller achieved a mean estimated closure error of 1.18±0.96cm, an absolute orientation residual of 1.41±1.02°, and a cross-track geometric RMSE of 2.46±0.36cm relative to the nominal contour. In the virtual V16 campaign (N=10), all ten runs completed successfully, yielding an EKF position RMSE of 1.70±0.55cm and yaw RMSE of 0.97±0.24° against PhysX rigid-body ground truth (${\mathrm{GT}}_{\mathrm{sim}}$).

Crucially, Stage 1 exposed the inherent optimism of purely proprioceptive state estimation. While the virtual EKF reported an internal closure error of 0.95±0.17cm, PhysX ground truth logged an actual terminal displacement of 3.39±0.90cm (2.44cm underestimation). The Stage 1 results establish a closed-loop waypoint repeatability and EKF-consistency baseline; metrics are restricted strictly to step-tracking geometry and internal filter consistency.

### 4.2. Stage 2: Continuous Turning Dynamics, Loop Asymmetry, and Proprioceptive Optimism

Stage 2 subjected the UGV to continuous, unsegmented turning dynamics over a nominal R=0.50m figure-eight trajectory. Unlike the square test, this maneuver avoids discrete $90^{\circ }$ stops and therefore exposes continuous heading accumulation, direction-dependent wheel-ground interaction, caster effects, and estimator drift during alternating counter-clockwise (CCW) and clockwise (CW) motion.

In physical trials (n=10), execution at v=0.08m/s and nominal |ω|=0.16rad/s yielded a final absolute heading drift of 1.42±0.22°, cumulative rotational discrepancy of $D_{\mathrm{rot}}=5.29\pm 0.67$°, onboard EKF closure of 6.90±0.73cm, and an independently measured physical floor closure of 11.59±3.14cm.

The physical figure-eight trials exhibited a repeatable direction-dependent geometric asymmetry, with a mean counter-clockwise loop diameter of 1.0795m and a clockwise diameter of 1.1280m. Diagnostic analysis of the recorded command, IMU, and wheel-odometry signals indicated that this asymmetry cannot be attributed to the fused EKF yaw estimate alone. The measured IMU yaw-rate magnitudes were nearly symmetric between rotation directions, whereas the controller required a substantially larger angular-command magnitude during clockwise motion and the wheel-odometry response was strongly direction-dependent. These observations are consistent with residual left–right actuator, drivetrain, wheel–ground interaction, and low-speed deadband asymmetries. The physical motor driver already incorporated a static right-side PWM scaling factor; however, this compensation did not fully eliminate the direction-dependent response. The present experiments therefore identify the effect at the system level but do not isolate a single mechanical cause.

The virtual V17 figure-eight campaign reproduced stable closed-loop execution and allowed independent evaluation against /ground_truth/odom; however, exact physical-digital equivalence was not asserted, as physical CW loop expansion and physical endpoint displacement exceeded simulated values.

A central cross-domain finding is the existence of a highly consistent proprioceptive estimator-optimism gap across both domains:

- Physical Domain: Independent floor measurement gave an actual closure error of 11.59±3.14cm, whereas the onboard EKF reported 6.90±0.73cm (a physical optimism gap of 4.69±2.82cm).
- Virtual Domain: PhysX ground truth reported an actual closure error of 7.08±0.41cm, while the virtual EKF reported 1.92±0.10cm (a virtual optimism gap of 5.16±0.41cm).

The close agreement between the physical (4.69cm) and virtual (5.16cm) estimator-optimism gaps ($\Delta_{\mathrm{cross}}=0.47\;\mathrm{cm}$) indicates that the calibrated digital twin reproduces a comparable estimator-optimism tendency under continuous rotation. However, remaining differences in loop diameter and endpoint displacement confirm that exact physical–digital equivalence was not achieved.

### 4.3. Stage 3: Exteroceptive SLAM, Local Costmap Reactivity, and Safety Metrics

Stage 3 evaluated integrated autonomous navigation using 2D LiDAR-based SLAM (slam_toolbox), AMCL pose tracking, Nav2 global planning, and DWB local planner control (dwb_core::DWBLocalPlanner). Unlike Stages 1 and 2, which primarily evaluated proprioceptive wheel-odometry and IMU-based state estimation without exteroceptive localization, Stage 3 evaluated LiDAR-supported localization, local costmap reactivity, goal convergence, obstacle clearance, collision count, and recovery behavior.

Both physical (n=10) and virtual (N=10) Stage 3 campaigns used matched start-to-goal corridors, identical dynamic-cylinder geometries ($r_{c}=0.125\;m$ positioned at $x_{c}=0.6091\;m$), and identical DWB local-planner parameters, including a 0.16m footprint, 0.25m inflation radius, and 0.25m goal tolerance. Across the official validation trials (R06–R15), both domains achieved 100% goal completion ($P_{\mathrm{success}}=1.0$), with zero physical contact events ($C_{\mathrm{count}}=0$). These outcomes are summarized using the safety-performance vector defined in Equation (16).

The Stage 3 results demonstrate that exteroceptive sensing and localization constrain cumulative position drift and reduce dependence on purely proprioceptive dead reckoning. Terminal goal-error magnitudes were numerically similar (0.211±0.011m for physical AMCL and 0.212±0.030m for virtual ground truth, with $\Delta_{\mathrm{cross}}=0.001\;m$) and remained within the Nav2 goal-checker tolerance. This result should be interpreted as similarity in terminal navigation behavior rather than equivalence in absolute localization. Domain-specific differences remained in path clearance ($d_{\min}=0.122\;m$ physical versus 0.074m virtual) and navigation duration (22.405s physical versus 13.317s virtual median), reflecting physical motor-acceleration limits, approximately 50ms command-transport latency, and domain-dependent wheel–surface interaction not fully reproduced by the nominal simulation configuration. By isolating the three experimental stages, this discussion avoids mixing square-path closure metrics, figure-eight rotational dynamics, and maze-navigation safety vectors, thereby providing a clear, stage-specific evaluation of physical and simulated mobile-robot performance.

The local sensitivity campaign further shows that Stage 3 navigation robustness is parameter-dependent and asymmetric around the tested operating point. A +5% wheel-radius perturbation generated the largest systematic trajectory displacement, whereas a −5% wheel-separation perturbation produced the strongest operational instability, including an approximately fourfold increase in median navigation time and one aborted trial. In contrast, the tested ±10% PhysX damping variations remained comparatively close to the baseline response. The asymmetric outcomes for equal positive and negative perturbation magnitudes indicate a nonlinear closed-loop interaction among effective differential-drive geometry, localization, costmap evolution, and DWB replanning rather than a simple proportional sensitivity to parameter magnitude.

The complementary robustness and generalization campaign further indicates that the calibrated Stage 3 configuration maintained task completion under the tested obstacle-layout, surface, and LiDAR perturbations. Lateral obstacle displacement changed the selected avoidance geometry and consequently the path length and execution time, whereas the controlled low-friction condition remained close to the nominal baseline. LiDAR degradation produced the most pronounced increase in navigation-time variability and recovery activity without preventing task completion. These results support robustness within the evaluated perturbation set while avoiding extrapolation to untested environmental or sensor-degradation levels.

The generalization demonstrated here should therefore be interpreted at two levels. First, at the system level, the calibrated Jackson digital twin preserved successful closed-loop navigation under controlled changes in obstacle location, wheel–surface interaction, and LiDAR quality, indicating that its nominal behavior was not restricted to a single deterministic scenario. Second, at the methodological level, the proposed Real-to-Sim workflow is transferable to other differential-drive UGVs because it does not depend on Jackson-specific numerical values: the same procedure can be applied by independently measuring the physical geometry, documenting runtime odometry and sensor parameters, identifying simulator-specific effective parameters, and repeating the staged validation protocol. However, the numerical calibration parameters obtained for Jackson should not be transferred directly to another platform, and broader generalization claims would require additional validation under different obstacle geometries, surface materials, sensor-failure modes, payload conditions, and operating environments.

### 4.4. Limitations and Prospective Extensions

The present architecture uses an EKF to fuse wheel-odometry and IMU measurements, whereas LiDAR-based SLAM and AMCL provide exteroceptive correction at the localization and navigation level rather than being fused directly by the EKF. Although this arrangement reduces dependence on dead reckoning, it does not constitute a fault-tolerant estimation system because sensor faults are neither explicitly detected nor isolated. Recent approaches have addressed this limitation through redundant inertial sensing, statistical fault diagnosis, health-dependent measurement selection, and residual-based fault detection and exclusion [24,25,26]. In particular, Xu et al. combined parity-vector fault diagnosis with a generalized likelihood-ratio test and an initial-alignment procedure comprising analytical coarse alignment followed by UKF-based fine alignment under large attitude errors [24]. For mobile robots, Kheirandish et al. developed a fault-tolerant fusion architecture based on multiple-model Kalman filtering to detect and accommodate bias, drift, and oscillatory sensor faults [27]. A fault-tolerant extension of Jackson should therefore investigate innovation monitoring, adaptive measurement covariances, sensor exclusion, and degraded operating modes under controlled encoder, IMU, and LiDAR faults.

Learning-based robust control constitutes a second extension of the calibrated digital-twin framework. The experiments presented here concern a single UGV controlled through conventional waypoint and Nav2 navigation components; consequently, they do not demonstrate learning-based robustness, multi-robot formation control, or coordinated UGV platooning. Recent work on UGV formations has combined proximal policy optimization and imitation learning with a belief-state encoder that integrates visual and proprioceptive information, improving simulated formation control under sensor noise and visual occlusion [28]. Complementarily, Amokrane et al. combined deep deterministic policy gradients with adaptive active disturbance rejection control for leader–follower coordination of unmanned tracked vehicles, improving simulated tracking robustness under track slippage, high-frequency measurement noise, and varying leader velocities [29]. These approaches are relevant to future coordinated Jackson platoons because they address uncertain perception, external disturbances, and nonlinear interactions that are difficult to represent using fixed control laws. However, extending the present study to a platoon would require additional modeling and validation of inter-vehicle communication, delay and packet loss, relative-pose uncertainty, collision constraints, and formation stability. The calibrated digital twin developed here provides a possible starting environment for this work, but learning-based policies would require independent training, explicit safety constraints, and physical transfer experiments before robust multi-UGV operation could be claimed.

Finally, the current navigation experiments use 2D LiDAR, while the two onboard cameras were not employed for localization, semantic segmentation, or obstacle avoidance. Semantic perception could complement the geometric information provided by LiDAR by distinguishing traversable surfaces, vegetation, structural boundaries, and obstacle classes. Recent studies have investigated monocular navigation using semantic images as inputs to DRL policies [30] and synthetic off-road datasets for improving real-world UGV segmentation [31]. Future integration into Jackson should evaluate camera calibration, illumination sensitivity, onboard inference latency, synthetic-to-real domain shift, and camera–LiDAR fusion before vision-based perception is incorporated into the safety-critical navigation loop.

## 5. Conclusions

This work presented a systematic Real-to-Sim parameter-identification protocol and a multi-stage cross-domain validation framework for the low-cost Jackson unmanned ground vehicle (UGV) and its NVIDIA Isaac Sim 4.5.0 digital twin. By distinguishing initial product/design references, directly measured physical geometry, empirically adjusted odometry/runtime parameters, and simulation-specific effective parameters, the study provides a reproducible methodology for analyzing the gap between physical execution and digital-twin behavior.

The experimental validation across three distinct testing stages demonstrated the following key findings:

- Stage 1 (Square Waypoint Repeatability): Closed-loop square-path execution over a 1.0m×1.0m trajectory demonstrated repeatable low-level motion behavior in both physical and virtual domains. The square experiment isolated discrete linear segments, approximately $90^{\circ }$ turns, endpoint closure, yaw convergence, and EKF consistency. In the virtual domain, comparison against PhysX ground truth (${\mathrm{GT}}_{\mathrm{sim}}$) showed that the EKF remained precise and repeatable but underestimated terminal displacement, illustrating the estimator-optimism effect associated with proprioceptive state estimation.
- Stage 2 (Continuous Dynamics and Estimator Optimism): Continuous figure-eight execution over nominal R=0.50m loops exposed direction-dependent physical turning asymmetry and estimator optimism. The physical platform completed all ten trials, with measured CCW and CW loop diameters of 1.0795±0.0167m and 1.1280±0.0322m, respectively. Independent manual floor measurements yielded a physical endpoint closure of 11.59±3.14cm, whereas the onboard EKF reported 6.90±0.73cm. In simulation, PhysX ground truth reported 7.08±0.41cm closure, while the virtual EKF reported 1.92±0.10cm. These results show that both domains exhibit a comparable proprioceptive estimator-optimism tendency under continuous turning, while also confirming that exact physical–digital equivalence was not achieved.
- Stage 3 (Exteroceptive SLAM and Dynamic Avoidance): Integrating 2D LiDAR-based mapping/localization, AMCL pose tracking, and Nav2 costmap local planning with dwb_core::DWBLocalPlanner reduced dependence on purely proprioceptive dead reckoning during online dynamic obstacle avoidance. Across the official physical and virtual validation campaigns (n=10 physical and N=10 virtual), both domains achieved goal completion without observed collision events. The Stage 3 results are therefore best interpreted through a safety-performance vector including task success, collision count, minimum obstacle clearance, recovery behavior, navigation time, and terminal goal tolerance, rather than through a single absolute trajectory-error metric. The local sensitivity campaign further showed asymmetric and nonlinear closed-loop behavior, with effective wheel geometry influencing navigation robustness more strongly than the tested PhysX damping variations. In the complementary robustness and generalization campaign, all 75 virtual trials completed successfully under controlled changes in obstacle placement, wheel–ground friction, and LiDAR degradation. Among these conditions, degraded LiDAR produced the largest increase in navigation-time variability while preserving successful task completion.

Overall, the results demonstrate that low-cost multi-sensor UGVs can be evaluated through a reproducible Real-to-Sim validation pipeline combining direct physical measurement, odometry calibration, ROS 2 state estimation, Isaac Sim ground-truth logging, and staged trajectory experiments. The findings also show that onboard proprioceptive estimators may be internally repeatable while remaining optimistic with respect to externally observed or simulator-provided reference poses. The calibrated digital twin provides a useful and technically defensible simulation baseline for studying cross-domain navigation behavior, while remaining physical–digital discrepancies should be reported explicitly rather than hidden behind endpoint-only metrics.

Future work will build on the calibrated digital-twin architecture in three directions: fault-tolerant state estimation under controlled sensor degradation; learning-based navigation and coordinated multi-UGV control with safety-aware Sim-to-Real validation; and semantic, obstacle-aware visual navigation through the onboard cameras and camera–LiDAR fusion. These extensions require dedicated experimental validation and are not capabilities demonstrated in the present study.

## Figures and Tables

**Table 1:** Comparison of the present Real-to-Sim calibration and validation framework with representative related studies. “Not primary” indicates that the corresponding aspect was not a principal reported contribution of the cited study.

Study	Main Scope	Physical Parameter Basis	Simulator-Specific Calibration	Repeated Real–Sim Validation	Estimator/Reference Analysis	Sensitivity/Generalization	Main Distinction
Rivera et al. [19]	Gazebo/ROS multibody UGV modeling	Yes	Model/physics configuration	Not primary	Not primary	Not primary	Physics-based UGV modeling and ROS/Gazebo integration
Daza et al. [13]	Reality-gap analysis for MPC trajectory tracking	Yes	Reality-gap characterization	Yes	Trajectory-level comparison	Scenario-based	Quantification of real–simulation trajectory-tracking differences
Ruhe et al. [8]	Isaac Sim-based MPPI controller optimization	Physical validation	Controller tuning	Yes	Not primary	Controller-parameter optimization	Simulation-based optimization followed by physical validation
Ding et al. [6]	ROS 2/Isaac Sim digital-twin synchronization	Yes	DT synchronization	Yes	Pose synchronization	Not primary	Physical–virtual state synchronization for autonomous mobile robots
Salimpour et al. [11]	RL-based Sim-to-Real mobile-robot navigation	Physical validation	Policy-oriented	Yes	Navigation performance	Cross-platform generalization	Zero-shot Sim-to-Real navigation across simulated and real robots
Bergs et al. [23]	Digital-twin predictive fidelity for mobile-robot navigation	Yes	Robot/sensor alignment	Yes; repeated trials	Navigation uncertainty	Scenario generalization	Systematic physical–digital navigation fidelity assessment
Present study	Parameter-level Real-to-Sim calibration and cross-domain validation	Direct geometry measurement, encoder scaling, and sensor characterization	Explicit physical/runtime vs. effective virtual parameter separation	Yes; three experimental stages	Physical endpoint measurements, onboard estimators, and simulator ground truth	105-run OFAT sensitivity + 75-run obstacle/surface/LiDAR generalization	Combined parameter identification, estimator-aware validation, sensitivity, and controlled generalization

**Table 2:** EKF configuration used in the physical UGV experiments.

Parameter	Experimental Value
Filter implementation	robot_localization 3.5.4
Update frequency	30Hz
Sensor timeout	0.20s
Two-dimensional mode	true
Map frame	map
Odometry/world frame	odom
Robot base frame	base_footprint
Published transform	odom → base_footprint
Wheel-odometry topic	/wheel/odom
Wheel-odometry variables	$v_{x}$, $v_{y}$
IMU topic	/imu/data
IMU variable	$\omega_{z}$
Wheel-odometry queue size	10
IMU queue size	50
Differential modes	false for both inputs
Relative modes	false for both inputs
Nodelay modes	false for both inputs
Gravity removal	false
Dynamic process-noise covariance	false
$R_{v_{x}}$	$0.01\;{\left(m/s\right)}^{2}$
$R_{v_{y}}$	$0.001\;{\left(m/s\right)}^{2}$
$R_{\omega_{z}}$	$0.0004\;{\left(\mathrm{rad}/s\right)}^{2}$

Note: The active Stage 3 navigation parameter set used for the sensitivity experiment is reported separately in Section 2.5.6.

**Table 3:** System-identification parameter mapping across initial design references, documented physical runtime configuration, direct physical measurements and calibration evidence, and Isaac Sim V16/V17 digital-twin parameters.

Parameter	Initial/Baseline Reference	Physical Runtime	Physical Measurement/Calibration Evidence	Isaac Sim Twin (V16/V17)	Unit
Wheel Radius (*r*)	≈0.034; seller/product reference, subsequently superseded	0.033000	0.033000; directly measured from the ≈66 mm outer diameter of the installed wheels	0.031650	m
Track Width/Wheel Separation (*B*)	0.198000 baseline	0.198000	≈0.198; consistent with the $360^{\circ }$ rotation check using the measured r=0.033 m	0.210481	m
Encoder Resolution ($C_{e}$)	652.0	621.0	617.0 (L)/621.0 (R) calibration history; floor-translation scaling	N/A	TPR
Role/Context	Initial product/encoder reference	Documented active odometry configuration	Direct physical-geometry measurement and empirical encoder/rotation checks	Simulation-specific effective geometry	—
Odom Source	N/A	Wheel-odometry driver publishing /wheel/odom	Same physical wheel-odometry pipeline; evaluated against floor trials	OmniGraph joint-state odometry publisher	—
EKF Function	N/A	Fuses /wheel/odom and /imu/data	N/A	Fuses virtual odometry and filtered IMU	—
Gyro Orientation	Uncorrected sensor convention	Audited MPU-6050; REP-103-consistent yaw sign	Same as physical runtime	Synthetic virtual IMU; ROS-consistent convention	—
IMU Yaw Scale	1.000	1.000	1.000	0.6953	—
Joint Stiffness (*K*)	N/A	N/A	N/A	0.0	N·m/rad
Joint Damping (*D*)	N/A	N/A	N/A	3000.0	N·m·s/rad
Static Gravity (*g*)	9.810 reference	9.299 measured	9.810 corrected target	9.810	m/s^2^
Accelerometer Magnitude Scale	1.000 raw	1.000	1.054× scale factor	1.000	—

**Table 4:** Experimental design of the Stage 3 one-factor-at-a-time local parameter sensitivity analysis.

Condition	*r* (m)	*B* (m)	PhysX *D*	Perturbation	Runs
C00_BASE	0.03300	0.19800	10000	Baseline	15
C01_R_MINUS5	0.03135	0.19800	10000	r−5%	15
C02_R_PLUS5	0.03465	0.19800	10000	r+5%	15
C03_B_MINUS5	0.03300	0.18810	10000	B−5%	15
C04_B_PLUS5	0.03300	0.20790	10000	B+5%	15
C05_D_MINUS10	0.03300	0.19800	9000	D−10%	15
C06_D_PLUS10	0.03300	0.19800	11000	D+10%	15

**Table 5:** Comparative performance metrics across ten repeated physical trial distributions (n=10) for raw wheel odometry and EKF-assisted state feedback control.

Performance Metric	Raw Wheel Odometry	EKF-Assisted Loop
Mean Onboard Closure Error (cm)	36.35±13.90	1.18±0.96
Median Onboard Closure Error (cm)	31.11	1.06
Maximum Onboard Closure Error (cm)	65.35	3.21
Mean Absolute Final Heading Deviation (°)	25.97±10.21	1.41±1.02
Geometric RMSE Relative to Nominal Square (cm)	14.75±4.09	2.46±0.36
Estimated Trajectory Distance Length (m)	4.133±0.020	4.133±0.020

**Table 6:** Aggregate statistical performance metrics across ten continuous physical figure-eight trajectory trials (N=10).

Performance Metric	Mean ± SD	95% CI	Min.–Max. Range
Motion Duration (s)	96.42±2.07	[94.94, 97.91]	92.40–99.80
CCW Rotational Error (°)	1.94±0.25	[1.76, 2.12]	1.47–2.29
CW Rotational Error (°)	3.36±0.43	[3.05, 3.66]	2.38–3.75
Cumulative Rotational Discrepancy ($D_{\mathrm{rot}}$) (°)	5.29±0.67	[4.81, 5.77]	3.85–6.02
Absolute Final Heading Drift (°)	1.42±0.22	[1.26, 1.57]	0.91–1.70
EKF Odometry Closure Error (cm)	6.90±0.73	[6.38, 7.42]	6.14–8.58
Manually measured physical closure error (cm)	11.59±3.14	[9.34, 13.83]	6.32–17.03
Physical CCW Loop Diameter (cm)	107.95±1.67	[106.75, 109.15]	106.00–111.00
Physical CW Loop Diameter (cm)	112.80±3.22	[110.49, 115.11]	108.00–118.00
EKF Cross-Track RMSE (cm)	9.48±1.39	[8.49, 10.47]	7.55–12.13
EKF Reconstructed Path Length (m)	6.96±0.11	[6.89, 7.04]	6.84–7.22

**Table 7:** Comparative estimation error parameters between the fused EKF framework and unassisted raw wheel odometry (N=10).

Trajectory Error Metric	Fused EKF Baseline	Raw Wheel Odometry Only
Mean Terminal Closure Error (cm)	6.90±0.73	207.00±26.00
Cross-Track RMSE (cm)	9.48±1.39	117.00±11.00
Maximum Cross-Track Deviation (cm)	22.52±3.04	226.00±6.00
Heading-Error RMSE (°)	5.46±0.63	64.10±5.65

**Table 8:** Statistical trajectory metrics for the Jackson UGV digital twin across ten repeated simulated trials (N=10) in NVIDIA Isaac Sim 4.5.0 (V16 Campaign).

Performance Metric	Mean ± SD	95% CI	Min.–Max. Range
Wall-clock runtime (s)	94.97±4.69	[91.62, 98.33]	90.25–104.70
GT Path Length (m)	4.0024±0.0070	[3.9973, 4.0074]	3.9909–4.0151
EKF Path Length (m)	3.9877±0.0109	[3.9799, 3.9955]	3.9703–4.0048
GT Cumulative Rotation (°)	360.23±1.02	[359.50, 360.96]	358.31–361.52
EKF Cumulative Rotation (°)	359.47±0.54	[359.09, 359.85]	358.34–360.08
GT Endpoint Closure Error (cm)	3.39±0.90	[2.74, 4.03]	2.33–5.16
EKF Endpoint Closure Error (cm)	0.95±0.17	[0.83, 1.07]	0.78–1.24
EKF Position RMSE vs. GT (cm)	1.70±0.55	[1.31, 2.09]	0.68–2.46
EKF Yaw RMSE vs. GT (°)	0.97±0.24	[0.80, 1.14]	0.60–1.35
Cross-Track Position RMSE (cm)	2.93	—	Max: 5.14

**Table 9:** Local sensitivity of Stage 3 closed-loop navigation to effective wheel-geometry and PhysX damping perturbations. The parameter values associated with each condition are given in Table 4. Continuous variables are reported as median [IQR].

Condition	Perturbation	Completion	$D_{\mathrm{traj}}$ (cm)	$d_{\min}$ (cm)	$t_{\mathrm{nav}}$ (s)
C00_BASE	Baseline	15/15	1.05 [0.98, 2.22]	8.17 [6.94, 9.01]	13.23 [12.98, 13.55]
C01_R_MINUS5	r−5%	15/15	2.24 [1.25, 37.42]	8.35 [5.83, 9.52]	12.12 [12.02, 13.55]
C02_R_PLUS5	r+5%	15/15	35.80 [3.87, 36.89]	4.95 [4.52, 6.03]	25.20 [14.58, 58.15]
C03_B_MINUS5	B−5%	14/15	4.25 [3.45, 36.93]	5.65 [4.50, 6.37]	54.31 [17.75, 56.77]
C04_B_PLUS5	B+5%	15/15	1.05 [0.95, 1.17]	8.62 [7.95, 8.84]	12.37 [12.23, 12.85]
C05_D_MINUS10	D−10%	15/15	1.29 [1.03, 4.44]	6.90 [4.90, 8.55]	13.90 [12.91, 14.76]
C06_D_PLUS10	D+10%	15/15	1.17 [0.70, 2.70]	8.20 [6.89, 8.72]	13.30 [12.84, 13.90]

**Table 10:** Robustness and generalization evaluation of the Stage 3 digital twin under obstacle-layout, surface, and LiDAR perturbations. Continuous metrics are reported as median [IQR] over 15 trials per condition.

Condition	Controlled Variation	Success	Navigation Time (s)	GT Path Length (m)	Min. Clearance (cm)
C00 Baseline	Nominal Stage 3	15/15	13.23 [12.98, 13.55]	1.885 [1.870, 1.892]	8.17 [6.94, 9.01]
G01	Obstacle Y+0.15m	15/15	10.73 [10.55, 11.23]	1.758 [1.740, 1.770]	11.63 [10.75, 12.59]
G02	Obstacle Y−0.15m	15/15	9.12 [9.08, 9.30]	1.708 [1.688, 1.722]	7.40 [7.16, 7.76]
G03	Low-friction surface, $\mu_{s}=0.30$, $\mu_{d}=0.25$	15/15	12.33 [11.99, 12.68]	1.878 [1.874, 1.892]	8.61 [7.87, 9.24]
G04	LiDAR: σ=0.03m and 5% dropout	15/15	18.12 [13.23, 32.87]	1.917 [1.903, 1.934]	9.69 [7.89, 11.07]

**Table 11:** Summary of motion/navigation duration and simulator computational runtime across the three validation stages. Virtual motion duration is measured using Isaac Sim simulation time, whereas wall-clock runtime represents elapsed computational execution time.

Experimental Stage	Physical Motion/ Navigation Time (s)	Virtual Simulation Time (s)	Virtual Wall-Clock Runtime (s)
Stage 1—Square	47.52±0.26	67.17±0.15	94.97±4.69
Stage 2—Figure-Eight	96.43±2.07	98.26±0.04	Not separately recorded
Stage 3—Dynamic Obstacle	22.405±0.816	18.063±14.823 (median: 13.317)	Not separately recorded

**Table 12:** Cross-domain quantitative trajectory comparison between physical UGV execution (n=10) and calibrated Isaac Sim V16 digital twin simulation (n=10) for the 1m×1m square trajectory.

Metric	Physical UGV (EKF)	Digital Twin (Isaac Sim)	Cross-Domain Deviation (Δ)
Mean Estimated Closure Error (cm)	1.18±0.96	0.95±0.17	0.23cm
Ground-Truth Closure Error (cm)	—	3.39±0.90	—
Absolute Yaw Error (°)	1.41±1.02	0.97±0.24	0.44°
Geometric/Cross-Track RMSE (cm)	2.46±0.36	1.70±0.55	0.76cm
Estimated Path Length (m)	4.133±0.020	3.988±0.011	0.145m
Motion Duration (s)	47.52±0.26	67.17±0.15 (simulation time)	19.65s

**Table 13:** Quantitative cross-domain comparison between physical UGV execution (N=10) and calibrated Isaac Sim digital twin simulation (N=10) for the Stage 2 continuous figure-eight trajectory.

Performance Metric	Physical UGV	Isaac Sim Digital Twin	Cross-Domain Deviation (Δ)
CCW Loop Diameter (m)	1.0795±0.0167	1.0429(GT)/0.9882(EKF)	0.0366m(GT)
CW Loop Diameter (m)	1.1280±0.0322	1.0187(GT)/1.2368(EKF)	0.1093m(GT)
Actual Endpoint Closure (cm)	11.59±3.14(Floor)	7.08±0.41(PhysXGT)	4.51cm
EKF Estimated Closure (cm)	6.90±0.73	1.92±0.10	4.98cm
Estimator Optimism Gap (cm)	4.69±2.82	5.16±0.41	0.47cm
Final Absolute Heading Residual (°)	1.42±0.22	1.41±0.15	0.01°
Motion Duration (s)	96.43±2.07	98.26±0.04(SimTime)	1.83s
Trajectory Position RMSE vs. GT (cm)	—	8.63±0.40	—
Trajectory Yaw RMSE vs. GT (°)	—	4.79±0.29	—
Rotational Discrepancy $D_{\mathrm{rot}}$ (°)	5.29±0.67	6.50±0.43	1.21°
Translational Discrepancy $D_{\mathrm{trans}}$ (cm)	—	5.52±0.45	—

**Table 14:** Cross-domain SLAM mapping fidelity, dynamic obstacle navigation parameters, and safety vector ($S_{\mathrm{vector}}$) evaluation across official physical UGV trials (n=10, runs R06–R15) and calibrated Isaac Sim simulations (N=10, runs R06–R15) in the Stage 3 Maze Navigation.

Navigation and Safety Metric	Physical UGV (DWB Planner)	Isaac Sim Digital Twin (DWB Planner)	Descriptive Comparison (Δ/Note)
Task Completion Rate ($P_{\mathrm{success}}$)	100% (10/10)	100% (10/10)	Δ=0.0%
Total Collision Count ($C_{\mathrm{count}}$)	0	0	Δ=0
Dynamic Trigger Displacement (m)	0.122±0.007	0.122±0.001	Δ=0.000m
Navigation Time ($t_{\mathrm{nav}}$) (s)	22.405±0.816	18.063±14.823(Med:13.317)	Nav. time vs. physical motion
Reconstructed Path Length (m)	1.958±0.014	1.888±0.039(Med:1.877)	Δ=0.070m(Virtualshorter)
Trajectory Efficiency (η)	90.4±0.7%	93.7±1.9%	Δ=3.3%
Terminal Goal Posture Error (m)	0.211±0.011(AMCL)	0.212±0.030(GT)	Δ=0.001m
Minimum Obstacle Clearance ($d_{\min}$) (m)	0.122±0.007	0.074±0.016(Min:0.043)	Δ=0.048m(Virtualcloser)
Local Recovery Actions ($E_{\mathrm{recovery}}$)	0.0	1.200±1.687(Med:0.500)	Occurred primarily in R13
Global Route-Side Switches	0	2.900±7.810(Med:0.000)	25 switches in run R13
Map Structural Similarity (SSIM)	0.885±0.021	0.942±0.010	Δ=0.057

## Data Availability

The ROS 2 bag files, processed trajectory CSV files, Real-to-Sim calibration scripts, and plotting utilities used in this study are publicly available in the Zenodo repository at https://doi.org/10.5281/zenodo.21792138. Additional information is available from the corresponding author upon reasonable request.

## Abbreviations

     The following abbreviations are used in this manuscript:

AMCL	Adaptive Monte Carlo Localization
DWB	Dynamic Window Approach (DWB Local Planner)
EKF	Extended Kalman Filter
IMU	Inertial Measurement Unit
LiDAR	Light Detection and Ranging
QoS	Quality of Service
RMSE	Root-Mean-Square Error
ROS 2	Robot Operating System 2
SLAM	Simultaneous Localization and Mapping
SSIM	Structural Similarity Index Measure
UGV	Unmanned Ground Vehicle

## Appendix A. Detailed Physical System Identification and Calibration Derivations

This appendix documents the physical measurement and calibration procedures used to establish the Jackson UGV kinematic and odometric parameters summarized in Section 2.4. Particular care is taken to distinguish directly measured physical geometry from parameters adjusted through floor-based odometry calibration. An early wheel-radius value of approximately 0.034m originated from the seller/product specification used during the initial platform configuration; it was not identified from the experimental calibration procedure and was subsequently superseded by direct measurement of the installed wheels.

The simulation-specific Isaac Sim V16/V17 parameters are reported separately in Appendix B because they represent effective virtual parameters used within the PhysX-based digital twin and should not be interpreted as direct physical measurements of the Jackson UGV.

### Appendix A.1. Physical Wheel Geometry and Encoder-Scaling Calibration

The installed rubber drive wheels were measured manually using a measuring tape. The measured outer diameter was approximately

(A1) $$D_{\mathrm{wheel}}\approx 0.066\;m,$$

which gives the physical wheel radius

(A2) $$r_{\mathrm{meas}}=\frac{D_{\mathrm{wheel}}}{2}\approx 0.033\;m.$$

Accordingly, the documented physical ROS 2 wheel-odometry implementation used

(A3) $$r_{\mathrm{run}}=0.033\;m.$$

The previously referenced value of approximately 0.034m was an early seller/product specification and was not obtained from the straight-line calibration experiments. It is therefore treated only as a superseded historical reference and not as an experimentally identified physical wheel radius.

Straight-line floor experiments were subsequently used to calibrate the conversion from encoder counts to traveled distance while retaining the directly measured physical wheel radius. Let $N_{i}$ denote the accumulated encoder counts for wheel i∈{L,R} over a sampling interval or calibration trajectory. The corresponding distance contribution is

(A4) $$\Delta s_{i}=\frac{2\pi r_{\mathrm{run}}}{C_{e}}N_{i},\;i\in \left\{L,R\right\},$$

where $C_{e}$ is the encoder-resolution parameter expressed in ticks per revolution (TPR).

The initial encoder reference was

(A5) $$C_{e,0}=652\;\mathrm{TPR}.$$

Using this initial value, the straight-line floor experiments showed an approximately 4.75% underestimation of traveled distance. Rather than modifying the measured wheel geometry, the correction was applied through the encoder-resolution parameter. If $d_{\mathrm{odom},0}$ denotes the displacement reconstructed using $C_{e,0}$ and $d_{\mathrm{meas}}$ is the corresponding floor-measured displacement, the corrected encoder resolution can be expressed as

(A6) $$C_{e,\mathrm{cal}}=C_{e,0}\frac{d_{\mathrm{odom},0}}{d_{\mathrm{meas}}}.$$

For the observed displacement ratio

(A7) $$\frac{d_{\mathrm{odom},0}}{d_{\mathrm{meas}}}\approx 1-0.0475=0.9525,$$

the resulting encoder-resolution estimate is

(A8) $$C_{e,\mathrm{cal}}\approx 652\times 0.9525\approx 621\;\mathrm{TPR}.$$

The documented physical runtime configuration therefore retained

(A9) $$r_{\mathrm{run}}=0.033\;m$$

and applied the practical linear odometry correction through the active encoder-resolution parameter

(A10) $$C_{e,\mathrm{run}}=621\;\mathrm{TPR}.$$

The calibration history also contains side-specific estimates of approximately 617.0TPR for the left wheel and 621.0TPR for the right wheel. These values document the observed left–right encoder scaling behavior; the active runtime parameter used for the reported physical experiments was 621TPR.

### Appendix A.2. Wheel-Separation Consistency Check

The documented physical wheel-separation parameter used by the differential-drive odometry model was

(A11) $$B_{\mathrm{run}}=0.198\;m.$$

In-place rotation experiments provide an independent kinematic consistency check of this value. For a differential-drive platform, the relationship between the accumulated left- and right-wheel angular displacements and the measured chassis rotation is

(A12) $$B_{\mathrm{check}}=\frac{r_{\mathrm{meas}}\left(\Delta \phi_{R}-\Delta \phi_{L}\right)}{\Delta \theta_{\mathrm{meas}}},$$

where $\Delta \phi_{L}$ and $\Delta \phi_{R}$ denote the accumulated angular displacements of the left and right drive wheels, respectively, and $\Delta \theta_{\mathrm{meas}}$ is the measured absolute chassis rotation.

For the $360^{\circ }$ rotation experiment,

(A13) $$\Delta \theta_{\mathrm{meas}}=2\pi \;\mathrm{rad},$$

and the recorded wheel-displacement difference was

(A14) $$\Delta \phi_{R}-\Delta \phi_{L}=37.70\;\mathrm{rad}.$$

Using the directly measured physical wheel radius $r_{\mathrm{meas}}=0.033\;m$ gives

(A15) $$B_{\mathrm{check}}=\frac{0.033\times 37.70}{2\pi }\approx 0.198\;m.$$

Thus, the rotation experiment is consistent with the documented physical/runtime wheel separation $B_{\mathrm{run}}=0.198\;m$ and does not require a separate $B_{\mathrm{cal}}$ value.

In summary, the physical geometry used in the reported experiments is characterized by the directly measured wheel radius

(A16) $$r_{\mathrm{meas}}=r_{\mathrm{run}}=0.033\;m,$$

and the documented wheel separation

(A17) $$B_{\mathrm{run}}=0.198\;m.$$

Physical floor calibration was applied primarily through the encoder-resolution parameter, with an initial reference of 652TPR and an active runtime value of 621TPR. These physical quantities are distinct from the simulation-specific Isaac Sim V16/V17 effective parameters $r_{\mathrm{sim}}=0.031650\;m$ and $B_{\mathrm{sim}}=0.210481\;m$.

## Appendix B. System Identification and Kinematic Calibration Derivations

This appendix documents the empirical calibration history, mathematical derivations, and configuration parameters used to synchronize physical UGV execution with the NVIDIA Isaac Sim digital twin.

### Appendix B.1. Physical Floor Calibration and Tick-Rate Scaling

The physical odometry baseline used the directly measured wheel radius $r_{\mathrm{meas}}=0.033\;m$, the documented wheel separation $B_{\mathrm{run}}=0.198\;m$, and an initial uncalibrated encoder setting of 652TPR. These values defined the initial physical odometry configuration. During linear translation experiments across 1.0m to 3.0m test spans, the initial encoder configuration exhibited a systematic −4.75% linear displacement deficit. Rather than altering the directly measured physical wheel geometry, calibration was applied to the low-level differential-drive odometry conversion by adjusting the active ticks-per-revolution parameter:

(A18) $$\mathrm{meters}\_\mathrm{per}\_\mathrm{tick}=\frac{2\pi r_{\mathrm{run}}}{\mathrm{ticks}\_\mathrm{per}\_\mathrm{rev}}.$$

By adjusting the active encoder resolution parameter in hardware.yaml from 652.0TPR down to 621.0TPR (617.0TPR left/621.0TPR right values documented in the calibration history), the linear resolution per tick was increased by +4.99%. This software tick scaling compensates for tire deflection under payload, reducing physical linear odometry error to <0.009m over 1.20m translation without introducing geometric mismatch into URDF visualization models.

### Appendix B.2. Virtual Digital Twin System Identification (Isaac Sim V16)

In NVIDIA Isaac Sim 4.5.0, rigid-body contact solvers (PhysX) introduce simulated surface compliance and non-holonomic tread scrubbing during differential turns. The physical wheel geometry was established independently by direct measurement of the installed wheels and provided the mechanical and runtime reference for constructing the digital twin. The virtual values $r_{\mathrm{sim}}=0.031650\;m$ and $B_{\mathrm{sim}}=0.210481\;m$ were subsequently introduced as simulator-specific effective parameters during the pre-validation calibration of the V16/V17 virtual-odometry model. Their refinement was evaluated against the Isaac Sim rigid-body reference pose published on /ground_truth/odom to improve consistency among virtual joint-state odometry, EKF output, and simulated rigid-body motion. These parameters should therefore not be interpreted as additional physical measurements of the Jackson UGV, but as effective virtual parameters that compensate for simulator-specific contact and odometry behavior. The resulting virtual configuration, alongside the active physical runtime parameters, is summarized in Table A1.

The V16/V17 parameters summarized in Table A1 should not be confused with the active Stage 3 closed-loop navigation operating point used for the local sensitivity analysis, namely $r_{0}=0.03300\;m$, $B_{0}=0.19800\;m$, and $D_{0}=10,000$. The Stage 3 configuration and its controlled perturbations are defined separately in Section 2.5.6.

**Table A1:** Calibration history and parameter allocation across initial references, the physical ROS 2 runtime configuration, and the Isaac Sim V16/V17 digital twin.

Parameter/Category	InitialReference	PhysicalRuntime	Isaac SimV16/V17
Wheel radius (*r*)	≈0.034m; seller/product reference, subsequently superseded	0.033000m; directly measured from the ≈66mm installed-wheel diameter	0.031650m; simulator-specific effective value
Track width/wheel separation (*B*)	0.198000m; documented physical baseline	0.198000m; active physical/runtime value; consistent with the $360^{\circ }$ rotation check	0.210481m; simulator-specific effective value
Encoder resolution ($C_{e}$)	652.0TPR; initial uncalibrated setting	621.0TPR; active runtime value; 617.0/621.0TPR left/right calibration history	N/A; virtual odometry obtained from simulated joint states
Active odometry source	N/A	esp32_odom_imu; publishes /wheel/odom	OmniGraph joint-state odometry publisher
EKF subscription	N/A	/wheel/odom and /imu/data	Virtual odometry and filtered IMU
IMU yaw scaling factor	1.0000	1.0000	0.6953; simulator-specific empirical tuning
PhysX drive damping (*D*)	N/A	N/A; physical DC-motor actuation	3000.0Nms/rad
PhysX drive stiffness (*K*)	N/A	N/A; physical DC-motor actuation	0.0Nm/rad

Note: The approximately 0.034m wheel-radius value was an early seller/product reference and was not obtained from physical calibration. It was superseded by the directly measured physical wheel radius $r_{\mathrm{meas}}=0.033\;m$. Physical floor calibration was applied primarily through the encoder-resolution parameter, with the active runtime value set to 621TPR. The Isaac Sim V16/V17 values $r_{\mathrm{sim}}=0.031650\;m$ and $B_{\mathrm{sim}}=0.210481\;m$ are simulator-specific effective parameters. They should not be confused with the separate Stage 3 navigation operating point, which used $r_{0}=0.03300\;m$, $B_{0}=0.19800\;m$, and $D_{0}=10,000$.

## Appendix C. ROS 2 Quality of Service (QoS) Configuration Profiles

This appendix details the quality of service (QoS) profiles used across the ROS 2 Humble middleware infrastructure with the Fast DDS backend. The profiles were selected to support low-latency telemetry capture, bounded memory usage, and repeatable Real-to-Sim data logging during physical and virtual navigation experiments.

**Table A2:** Summary of ROS 2 QoS profile mappings across functional subsystems.

Topic/Subsystem	Reliability	Durability	History/Depth	Notes
Sensor data (/scan, /imu/data)	Best Effort	Volatile	Keep Last (depth=5)	Low-latency telemetry
Odometry (/wheel/odom, /odometry/filtered)	Reliable	Volatile	Keep Last (depth=10)	State-estimation input/output
Velocity commands (/cmd_vel)	Reliable	Volatile	Keep Last (depth=1)	Application watchdog timeout: 0.60s
Dynamic transforms (/tf)	Reliable	Volatile	Keep Last (depth=100)	Runtime frame updates
Static transforms (/tf_static)	Reliable	Transient Local	Keep Last (depth=1)	Latched static frame tree
Map/localization state	Reliable	Transient Local	Keep Last (depth=1)	Static map and retained localization metadata

Fast DDS QoS profiles were configured to reduce frame drops during high-frequency /scan publication and multi-sensor telemetry aggregation. In addition, bounded history depths were used to limit memory pressure during edge execution on the NVIDIA Jetson Orin Nano. The 0.60s velocity-command timeout is treated as an application-level safety watchdog rather than as a ROS 2 QoS deadline parameter.

## Appendix D. Occupancy Grid Structural Similarity and Safety Metrics

### Appendix D.1. Map Structural Similarity Index (SSIM)

Cross-domain mapping fidelity between virtual ($M_{\mathrm{sim}}$) and physical ($M_{\mathrm{real}}$) rasterized occupancy grids is evaluated via

(A19) $$\mathrm{SSIM}\left(M_{\mathrm{sim}},M_{\mathrm{real}}\right)=\frac{\left(2\mu_{\mathrm{sim}}\mu_{\mathrm{real}}+C_{1}\right)\left(2\sigma_{\mathrm{sim},\mathrm{real}}+C_{2}\right)}{\left(\mu_{\mathrm{sim}}^{2}+\mu_{\mathrm{real}}^{2}+C_{1}\right)\left(\sigma_{\mathrm{sim}}^{2}+\sigma_{\mathrm{real}}^{2}+C_{2}\right)}$$

where $\mu_{i}$ and $\sigma_{i}^{2}$ denote localized spatial mean and variance parameters, and $C_{1}={\left(0.01\cdot L\right)}^{2}$, $C_{2}={\left(0.03\cdot L\right)}^{2}$ are dynamic range stabilization constants (L=255).

### Appendix D.2. Safety Evaluation Performance Vector

System safety behavior during obstacle course navigation is quantified via

(A20) $$S_{\mathrm{vector}}=\left[P_{\mathrm{success}},C_{\mathrm{count}},d_{\min},E_{\mathrm{recovery}},t_{\mathrm{nav}}\right]$$

where $P_{\mathrm{success}}$ is trial completion rate, $C_{\mathrm{count}}$ is collision count, $d_{\min}$ is minimum clearance to obstacles (m), $E_{\mathrm{recovery}}$ is the count of localized recovery rotations, and $t_{\mathrm{nav}}$ is the navigation time measured from the NavigateToPose feedback.

### Appendix D.3. Stage 3 Conservative Collision Envelope

For reproducibility of the Stage 3 minimum-clearance calculation, the conservative planar collision envelope was extracted from the audited Stage 3 USD geometry and expressed in the robot reference frame. Its axis-aligned bounds were

(A21) $$\begin{array}{cc} x & \in [-0.135000,\;0.135177]\;m,\\ y & \in [-0.135439,\;0.135000]\;m. \end{array}$$

These bounds define the conservative oriented collision envelope used for the geometric clearance metric. They represent an audited safety envelope of the Stage 3 model and should not be interpreted as an exact high-fidelity mesh-to-mesh distance or as a geometrically resized wheel model.
